# Defective Desmosomal Adhesion Causes Arrhythmogenic Cardiomyopathy by Involving an Integrin-αVβ6/TGF-β Signaling Cascade

**DOI:** 10.1161/CIRCULATIONAHA.121.057329

**Published:** 2022-10-21

**Authors:** Camilla Schinner, Lifen Xu, Henriette Franz, Aude Zimmermann, Marie-Therès Wanuske, Maitreyi Rathod, Pauline Hanns, Florian Geier, Pawel Pelczar, Yan Liang, Vera Lorenz, Chiara Stüdle, Piotr I. Maly, Silke Kauferstein, Britt M. Beckmann, Farah Sheikh, Gabriela M. Kuster, Volker Spindler

**Affiliations:** Department of Biomedicine, Section Anatomy (C. Schinner, H.F., A.Z., M.-T.W., M.R., P.H., C. Stüdle, P.I.M., V.S.), University of Basel, Switzerland.; Center for Transgenic Models (P.P.), University of Basel, Switzerland.; Department of Biomedicine, University Hospital Basel and University of Basel, Switzerland (L.X., V.L., G.M.K.).; Department of Biomedicine, Bioinformatics Core Facility (F.G.), University Hospital Basel, Switzerland.; Division of Cardiology (G.M.K.), University Hospital Basel, Switzerland.; Swiss Institute of Bioinformatics, Basel, Switzerland (F.G.).; Department of Medicine, University of California San Diego (Y.L., F.S.).; Department of Legal Medicine, University Hospital Frankfurt, Goethe University, Frankfurt am Main, Germany (S.K., B.M.B.).; Department of Medicine I, University Hospital, LMU Munich, Germany (B.M.B.).

**Keywords:** cardiomyopathies, desmosomes, fibrosis, integrins, intercalated disc, transforming growth factors

## Abstract

Arrhythmogenic cardiomyopathy (ACM) is characterized by progressive loss of cardiomyocytes with fibrofatty tissue replacement, systolic dysfunction, and life-threatening arrhythmias. A substantial proportion of ACM is caused by mutations in genes of the desmosomal cell–cell adhesion complex, but the underlying mechanisms are not well understood. In the current study, we investigated the relevance of defective desmosomal adhesion for ACM development and progression.

**Methods:**

We mutated the binding site of DSG2 (desmoglein-2), a crucial desmosomal adhesion molecule in cardiomyocytes. This DSG2-W2A mutation abrogates the tryptophan swap, a central interaction mechanism of DSG2 on the basis of structural data. Impaired adhesive function of DSG2-W2A was confirmed by cell–cell dissociation assays and force spectroscopy measurements by atomic force microscopy. The DSG2-W2A knock-in mouse model was analyzed by echocardiography, ECG, and histologic and biomolecular techniques including RNA sequencing and transmission electron and superresolution microscopy. The results were compared with ACM patient samples, and their relevance was confirmed in vivo and in cardiac slice cultures by inhibitor studies applying the small molecule EMD527040 or an inhibitory integrin-αVβ6 antibody.

**Results:**

The DSG2-W2A mutation impaired binding on molecular level and compromised intercellular adhesive function. Mice bearing this mutation develop a severe cardiac phenotype recalling the characteristics of ACM, including cardiac fibrosis, impaired systolic function, and arrhythmia. A comparison of the transcriptome of mutant mice with ACM patient data suggested deregulated integrin-αVβ6 and subsequent transforming growth factor–β signaling as driver of cardiac fibrosis. Blocking integrin-αVβ6 led to reduced expression of profibrotic markers and reduced fibrosis formation in mutant animals in vivo.

**Conclusions:**

We show that disruption of desmosomal adhesion is sufficient to induce a phenotype that fulfils the clinical criteria to establish the diagnosis of ACM, confirming the dysfunctional adhesion hypothesis. Deregulation of integrin-αVβ6 and transforming growth factor–β signaling was identified as a central step toward fibrosis. A pilot in vivo drug test revealed this pathway as a promising target to ameliorate fibrosis. This highlights the value of this model to discern mechanisms of cardiac fibrosis and to identify and test novel treatment options for ACM.

**Clinical Perspective**

**What Is New?**

- The tryptophan residue at position 2 of the desmosomal adhesion molecule DSG2 (desmoglein-2) is central for its binding function and cell–cell adhesion in vitro and in vivo.
- Mice with abrogated DSG2 binding function (DSG2-W2A) develop a cardiac phenotype recalling arrhythmogenic cardiomyopathy with fibrosis, impaired systolic function, ECG abnormalities, and ventricular arrhythmia.
- Increased integrin-αVβ6–dependent transforming growth factor–β signaling was identified as a driver of fibrosis.

**What Are the Clinical Implications?**

- We provide a new mouse model reproducing major features of arrhythmogenic cardiomyopathy that can be applied to study disease mechanisms and test therapeutic approaches.
- The model suggests that loss of mechanical cardiomyocyte coupling is a central and early event causing arrhythmogenic cardiomyopathy.
- Our pilot in vivo study highlights the applicability and potential to target integrin-αVβ6–dependent transforming growth factor–β release as new therapeutic approach to diminish the development of fibrosis in arrhythmogenic cardiomyopathy.

Patients with arrhythmogenic cardiomyopathy (ACM) present with features ranging from impaired cardiac function and ventricular arrhythmia to sudden cardiac death. This inherited disease, with a prevalence of 1:1000 to 1:5000, typically becomes evident in young adulthood. The therapeutic options for ACM—such as β-adrenergic receptor blockers, restriction of physical exercise, implantation of a cardiac defibrillator, or transplantation as ultima ratio—are limited to symptoms management.^1^ ACM is characterized by progressive loss of cardiomyocytes, cardiac fibrosis with fibrofatty tissue replacement, and ventricular dilatation. In the majority of cases, mutations in components of the desmosomal complex are causative for ACM.^1,2^

In cardiomyocytes, this cell–cell adhesion complex is composed of the desmosomal adhesion molecules DSG2 (desmoglein-2) and DSC2 (desmocollin-2), which are linked to desmin intermediate filaments by PG (plakoglobin), PKP2 (plakophilin-2), and DSP (desmoplakin).^3^ These components are an integral part of the intercalated disc (ICD), where they form a functional unit with molecules of adherens junctions, gap junctions, and others such as sodium channels.^4^ The ICD is essential to provide mechanical and electrical coupling to adjacent cardiomyocytes, which is a basis for coordinated contraction and function of the heart. In ACM, a variety of mutations in genes encoding for desmosomal molecules were described.^2^ Together with the finding of disrupted ICD ultrastructure,^5^ this formed the hypothesis of impaired cardiomyocyte cohesion as central step in ACM pathogenesis. However, data on the role of cell–cell adhesion are conflicting, and the effect of disrupted mechanical coupling for disease induction and progression is not resolved.^6–8^

In this study, we address the role of disrupted desmosomal adhesion in ACM development by interfering with the binding mechanism of DSG2. The crystal structure of DSG2 suggests the extracellular *trans*-interaction of desmosomal adhesion molecules to be mediated by a so-called tryptophan swap.^9^ Here, a tryptophan residue at position 2 is inserting into a hydrophobic pocket of the first extracellular domain of the respective molecule from the opposing cell and vice versa. Mutations of this tryptophan (ClinVar databank: VCV000199796, VCV000044282, VCV000420241)^10^ as well as of residues being part of the corresponding hydrophobic binding pocket (VCV000518623, VCV000925370, VCV001345680, VCV000044316) ^9,10^ were reported in patients with ACM.

To interfere profoundly with this mechanism, we inserted a mutation substituting the tryptophan-2 with an alanine (DSG2-W2A) and characterized its functional implication in vitro, in cultivated cells, and in a knock-in mouse model. We applied this in vivo model to identify mechanisms underlying ACM, aiming for new therapeutic approaches.

## Methods

Detailed methods are available in the Expanded Methods in the Supplemental Material.

### Human Heart Samples

Heart samples of patients with ACM and healthy controls were derived from forensic autopsy. This study was conducted according to the tenets of the Declaration of Helsinki and approved by the local ethics committees (license number 494-16 from LMU Munich and license number 152/15 from Goethe University). Samples were fixed and embedded in paraffin according to standard procedures.

### Animal Experiments

Mouse experiments were approved by the Cantonal Veterinary Office of Basel-Stadt (license numbers 2973_32878 and 3070_32419). Mice were housed under specific pathogen-free conditions with standard chow and bedding with a 12-hour day/night cycle according to institutional guidelines. Animals of both sexes were applied without bias. For inhibitor treatments, mice from the same litter and sex were paired and allocated randomly to the groups.

### Statistics and Data Compilation

Figures were compiled with Adobe Photoshop CC 2017 and Adobe Illustrator CC 2017. Statistical computations were performed with Prism 8 (GraphPad Software). For comparison of 2 or multiple groups, distribution of data was analyzed by a Shapiro-Wilk normality test, and group variances were analyzed by an *F*-test or a Brown-Forsythe test, respectively. According to the results of these tests, a parametric or nonparametric test with or without Welch correction for unequal variances was applied. For Kaplan-Meier survival analysis, significance was assessed by the Gehan-Breslow-Wilcoxon test. The statistical test used to compare the respective data sets is described in the corresponding figure legend. Statistical significance was assumed at *P*<0.05. Unless otherwise stated, data are presented as dot blot, with each dot representing the mean of the respective technical replicates of 1 biological replicate. Each animal or independent seeding of cells was taken as biological replicate, or as indicated in the legend. The mean value of these dots is shown as bar diagram ± SD.

### Data Availability

RNA sequencing data were deposited in the public database GEO (

accession number GSE181868). Additional data pertaining to the current article are available from the corresponding author upon request.

## Results

### W2A Mutation Abrogates DSG2 Interactions and Impairs Cell–Cell Adhesion In Vitro and In Vivo

**Figure 1.**
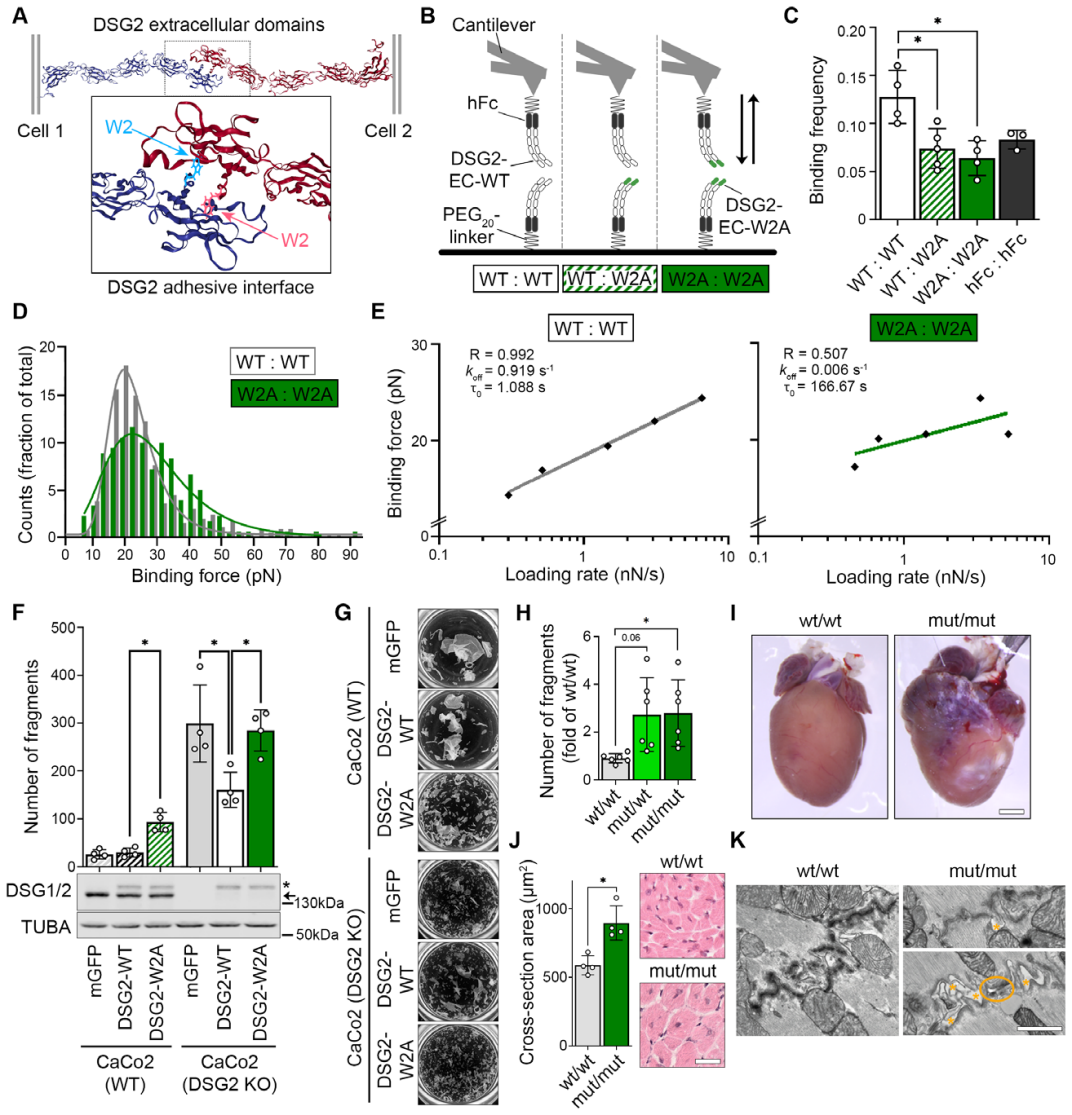
**DSG2 (desmoglein-2) adhesion is mediated by tryptophan swap at position 2. A**, Predicted interaction model of DSG2 extracellular domains by exchange of tryptophan residue at position 2 into a hydrophobic pocket of the opposing molecule. Cartoon 3D presentation of Protein Data Bank entry 5ERD^9^; tryptophan-2 is highlighted by ball and stick presentation. **B**, Schematic of single molecule force spectroscopy experiments. Recombinant extracellular domains (ECs) of wild-type DSG2 (DSG2-WT) or mutant DSG2 (DSG2-W2A) protein were coupled to a mica surface and atomic force microscopy cantilever by mean of a human Fc-tag (hFc) and a polyethylene glycol (PEG)_20_ linker and probed as indicated. **C**, Binding frequency of DSG2-W2A/DSG2-WT heterotypic and homotypic interactions at a pulling speed of 2 µm/s are shown. hFc served as control for nonspecific binding. *P<0.05, 1-way analysis of variance, Dunnett post hoc test. Each independent coating procedure with minimum 625 force curves is taken as biological replicate. **D**, Histogram of binding forces distribution with peak fit at a pulling speed of 2 µm/s corresponding to data in **C**. **E**, Determination of the bond half lifetime by the Bell equation^11^ of mean loading rates and binding forces analyzed from data of pulling speeds at 0.5, 1, 2, 5, and 7.5 µm/s. Average of values from 4 independent coating procedures with minimum 625 force curves each. R=R^2^, *k*_off_ =off-rate constant, τ_0_=bond half lifetime under zero force. **F**, Dissociation assays to determine cell–cell adhesion were performed in CaCo2 cells (WT or DSG2 KO background) expressing DSG2-WT-mGFP or DSG2-W2A-mGFP constructs. mGFP empty vector served as control. *P<0.05, 1-way analysis of variance, Sidak post hoc test. Corresponding Western blot analysis confirmed effective expression of DSG2 constructs (*) versus the endogenous protein (arrow) in CaCo2 cells. α-tubulin (TUBA) served as loading control. **G**, Representative images of monolayer fragmentation from experiments in **F**. **H**, Dissociation assays in immortalized keratinocytes isolated from neonatal murine skin of the respective genotype. *P<0.05 or as indicated, Welch analysis of variance, Dunnett post hoc test. **I**, Macroscopic cardiac phenotype of DSG2-W2A mut/mut mice at age 15 weeks. **J**, Cardiac hypertrophy was analyzed as mean cross-sectional area of cardiomyocytes in hematoxylin & eosin–stained sections. Scale bar, 30 µm. *P<0.05, unpaired Student *t* test. **K**, Representative images of intercalated discs acquired by transmission electron microscopy, 3 mice per genotype. Orange asterisks mark intercellular widening, orange circle marks a ruptured junction. Scale bar, 1 µm.

To study the functional consequences of impaired DSG2 binding, we generated the W2A point mutation (DSG2-W2A) to abrogate the proposed interaction mechanism of the only desmoglein expressed in cardiomyocytes (Figure 1A). First, we investigated in a cell-free setup whether DSG2-W2A is indeed affecting the binding properties of DSG2 interaction by applying single molecule force spectroscopy. Here, molecules are coupled to the tip of an atomic force microscopy probe and the surface of a mica sheet by a polyethylene glycol linker. By measuring the deflection of the probe during repeated approach to and retraction from the surface, binding events can be assessed quantitatively. From these data, properties such as binding frequency, binding force, and bond half-life time can be determined. Constructs were generated for expression of wild-type (WT) and mutant (W2A) DSG2 extracellular domains fused to a human Fc fragment and tested for homotypic (WT:WT, W2A:W2A) and heterotypic (WT:W2A) interaction properties (Figure 1B). These experiments showed a significant reduction of the frequency of W2A:W2A as well as WT:W2A interactions compared with WT:WT (Figure 1C). The frequency of the remaining W2A bindings was comparable to probing Fc alone, which indicates that these are mostly nonspecific interactions. Homotypic WT interactions display a clear binding force peak, whereas the remaining W2A interaction forces were spread widely, again pointing to the nonspecificity of these interactions (Figure 1D). The lifetime of DSG2 interactions under zero force (τ_0_) was determined by fitting the binding forces detected at different loading rates,^11^ yielding τ_0_=1.088s with R=0.993. In contrast, no sufficient fit (R=0.509) was possible for W2A interactions (Figure 1E). Together, these data outline that the tryptophan swap is the major interaction mechanism of DSG2.

To determine the effect of the W2A mutation on a cellular level, DSG2-WT and DSG2-W2A constructs were stably expressed in the epithelial cell line CaCo2 either in a WT or DSG2-deficient background (CaCo2[WT] or CaCo2[DSG2 KO]).^12^ Cell–cell dissociation assays were performed, in which a confluent cell monolayer is detached and exposed to defined mechanical stress. Expression of DSG2-W2A in CaCo2(WT) cells significantly reduced intercellular adhesion as indicated by an increased number of fragments (Figure 1F and 1G). Whereas expressing DSG2-WT in the CaCo2(DSG2 KO) line significantly rescued the impaired intercellular adhesion in response to DSG2 loss, the expression of DSG2-W2A had no effect on fragment numbers.

Because these data demonstrate the DSG2 tryptophan swap to be the central adhesive mechanism, we next generated a CRISPR/Cas9-based knock-in mouse model for DSG2-W2A to investigate the consequences of impaired DSG2 adhesion in vivo (Figure S1). In line with the studies in human cells, dissociation assays in keratinocyte cell lines generated from DSG2-W2A pups revealed reduced cell–cell adhesion in heterozygous (mut/wt) and homozygous (mut/mut) mutants (Figure 1H). Moreover, DSG2-W2A mice presented with a pronounced cardiac phenotype. Adult mut/mut mice demonstrated ventricular deformation, fibrotic and calcified areas, and hypertrophic cardiomyocytes (Figure 1I and 1J). In line with reduced intercellular adhesion of cardiomyocytes, transmission electron microscopy revealed disturbed ICDs with widened intercellular space and occasionally completely ruptured junctions in mut/mut hearts (Figure 1K).

A cardiac phenotype was also detectable during embryogenesis. Here, mating of heterozygous mice revealed a reduction of mut/mut offspring to 2.8% compared with the expected Mendelian ratio of 25%. Embryo dissections showed loss of the mut/mut animals between E12 and E14 (Figure S2A). The mut/mut embryos at day E12.5 appeared macroscopically pale with accumulation of blood in the cardiac area although the heart was still beating (Figure S2B). Cells suggestive for blood precursors were detectable in the pericardial space (Figure S2C). This finding suggests a rupture of the cardiac wall leading to pericardial bleeding and loss of mut/mut animals.

Together, these in vitro and in vivo data outline an essential role of the DSG2 tryptophan swap for cell–cell adhesion and demonstrate severe pathologies associated with impaired DSG2 interaction.

### DSG2-W2A Mutant Mice Resemble the Phenotype of ACM

Given the cardiac affection in mutant mice, the notion that a majority of patients with ACM are reported with mutations in desmosomal genes,^2^ and that mutations specifically of W2 have already been associated with ACM,^10^ we examined homozygous and heterozygous mice including both sexes with regard to histologic and clinical ACM measures in an age range of 12 to 80 weeks.

**Figure 2.**
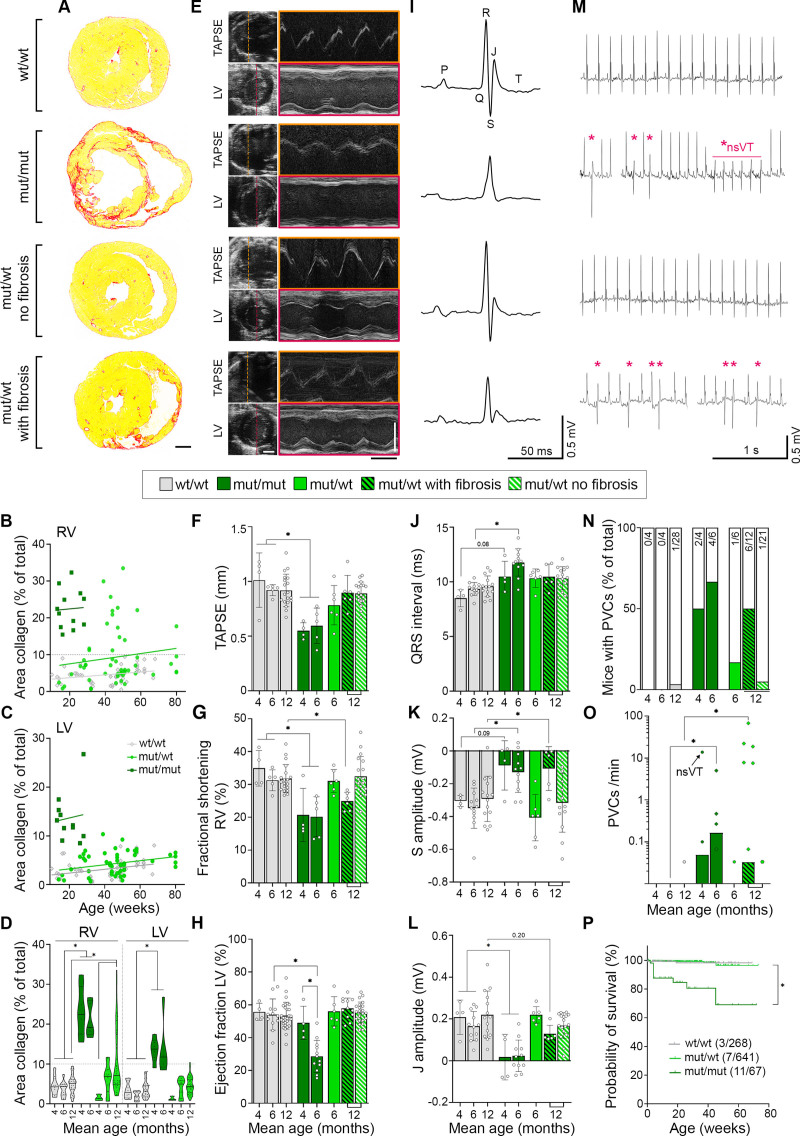
**DSG2 (desmoglein-2)–W2A mutant mice develop characteristics of arrhythmogenic cardiomyopathy. A**, Cardiac fibrosis detected by picrosirius red collagen staining with representative images of sections from 6-month-old DSG2-W2A mut/mut and 12-month-old wt/wt and mut/wt animals. Scale bar = 1 mm. **B** through **D**, Corresponding analysis of the area of collagen in the right ventricle (RV) and left ventricle (LV). Continuous lines indicate the simple linear regression between age and area of collagen. Hearts with >10% of collagen in the RV (gray dotted line) were defined as “with fibrosis.” Each dot represents 1 animal. *P<0.05, mixed effects analysis with LV and RV matched per animal, Sidak post hoc test. Lines indicate median and quartile values. **E**, Representative echocardiography images for measurements of the tricuspid annular plane systolic excursion (TAPSE) and LV function in 6-month-old mut/mut and 12-month-old wt/wt and mut/wt animals. Left side: 2D images from apical 4-chamber view for TAPSE and parasternal short axis view for LV. M-mode tracings on the right were performed along the line indicated on the left. Scale bars, white, 2 mm; black, 100 ms. Corresponding analysis of (**F**) TAPSE, *P<0.05, Kruskal-Wallis test with Dunn post hoc test; (**G**) fractional shortening of the RV, *P<0.05, Kruskal-Wallis test with Dunn post hoc test; and (**H**) ejection fraction of the LV, *P<0.05, 1-way analysis of variance, Sidak post hoc test. **I**, ECG recoded in lead II with representative curves from 6-month-old mut/mut and 12-month-old wt/wt and mut/wt animals. Definition of respective peaks is indicated in the wt/wt curve. Scale bars, vertical, 0.5 mV; horizontal, 50 ms. Corresponding analysis of (**J**) QRS interval, *P<0.05 or as indicated, Kruskal-Wallis test with Dunn post hoc test; (**K**) amplitude of the S peak, *P<0.05 or as indicated, 1-way analysis of variance, Sidak post hoc test; and (**L**) amplitude of the J peak (early repolarization), *P<0.05 or as indicated, Kruskal-Wallis test with Dunn post hoc test. **M**, Ventricular arrythmia detected by ECG during 30 minutes of baseline measurements with example curves from 6-month-old mut/mut and 12-month-old wt/wt and mut/wt animals. Asterisks mark premature ventricular contractions (PVCs); *nsVT indicates a nonsustained ventricular tachycardia detected in 1 mut/mut animal. Scale bars, vertical, 0.5 mV; horizontal, 1 s. Corresponding analysis of (**N**) percentage of mice presenting with PVCs. Values in bars indicate corresponding absolute number of mice with PVC (colored bars) compared with total number of mice evaluated (empty bar) and (**O**) PVC burden depicted as number of PVCs per minute, *P<0.05, Kruskal-Wallis test with Dunn post hoc test. The black arrow indicates the animal presenting with nonsustained ventricular tachycardia. **P**, Kaplan-Meier survival plot of DSG2-W2A mice from an analysis period of 3 years. Vertical lines indicate dropouts because of unrelated elimination (end of experiment, breeding, injuries). Values indicate corresponding absolute number of mice with sudden death compared with total number of mice evaluated. *P<0.05, Gehan-Breslow-Wilcoxon test. Box with color indications of respective groups in the middle applies to the entire figure.

Histologic analysis showed cardiac fibrosis, a major hallmark of ACM, in the myocardium of the right ventricle (RV) and left ventricle (LV) in mut/mut animals (Figure 2A through 2D), which did not profoundly increase over time. In contrast, heterozygous animals demonstrated a milder phenotype, with a fraction of animals (36%) starting to develop fibrosis in the RV around 6 months, whereas LV was unaffected. To better address differences over time, we pooled animals into groups with a mean age of 4 and 6 months for homozygous and 6 and 12 months for heterozygous mice and performed functional analyses by echocardiography and ECG parallel to histologic evaluation. Moreover, we separated 12-month-old heterozygous animals according to RV fibrosis into a group without and with fibrosis (>10% fibrotic area). Mut/mut animals showed impaired RV systolic function at both time points with reduced tricuspid annular plane systolic excursion as well as fractional shortening (Figure 2E through 2G and Table S1). LV systolic impairment with reduction of the ejection fraction and corresponding parameters worsened over time and became significant after 6 months, although no increase in fibrosis was detectable (Figure 2D and 2H and Table S1). In mut/wt mice, RV fractional shortening was significantly reduced in 12-month-old animals with fibrosis. In ECG recordings of mut/mut mice for 30 minutes under anaesthesia, we noted deformation of the QRS complex with elongated QRS interval and reduced amplitude of the S peak with effects more pronounced in the older group; heterozygous animals with RV fibrosis showed similar effects or trends (Figure 2I through 2K and Table S1). Because the T wave was not clearly detectable in mutants, the J peak amplitude as indicator for early repolarization was determined. This was reduced in the mut/mut groups with same trend for fibrotic mut/wt hearts (Figure 2L). Parallel to these depolarization and repolarization abnormalities, we also noted the occurrence of ventricular arrhythmia, ranging from single premature ventricular contractions (PVCs) to multiple, multifocal PVCs and nonsustained ventricular tachycardia in 1 mouse (Figure 2M through 2O). The fraction of homozygous and heterozygous animals with PVCs and the frequency of these events correlated with age and fibrosis. Moreover, a substantial fraction (16%) of mut/mut animals was found dead during the observation period, which strongly suggests the occurrence of malignant arrhythmia with sudden death (Figure 2P). Investigating sex-related differences, no effect was observed on the extent of fibrosis or echocardiographic parameters. However, in mut/wt mice, PVCs were found in males only and male mut/mut mice were more prone to premature sudden death compared with females (Figure S3).

In summary, mutant animals show histopathologic features with echocardiography and ECG abnormalities similar to patients with ACM. Within the limits of a murine model, both genotypes fulfil the Padua criteria^13^ to establish the diagnosis of ACM (Table S2). Here, DSG2-W2A mut/mut animals resemble the phenotype of biventricular ACM, whereas in mut/wt mice, the phenotype is milder with variable penetrance and age-dependent occurrence only in the RV. According to the Padua criteria,^13^ the heterozygous genotype recalls the characteristics of a right-dominant ventricular ACM, as long as fibrosis was present. Together, these data demonstrate that loss of desmosomal adhesion is sufficient to induce an ACM phenotype and that the DSG2-W2A model may resemble 2 different variants of the disease.

### Integrin-β6 Expression Is Altered in ACM Patient and DSG2-W2A Mouse Samples

Next, we applied this mouse model to investigate mechanisms leading to ACM. RNA sequencing was performed from ventricles of mut/mut and wt/wt hearts both before onset of fibrosis (age 5 days) and when the biventricular fibrosis and ACM phenotype was established (after 9 weeks). First, we compared sequencing data from 9-week-old mice with the top differentially expressed genes derived from published transcriptomic data sets of ACM patient hearts (GEO database: GSE107157/GSE107480^14^ and GSE29819^15^). Murine samples clustered according to their genotype and mut/mut hearts resembled the gene expression pattern of patients (Figure S4), further supporting the observation that mutant mice faithfully reproduce major aspects of the disease.

**Figure 3.**
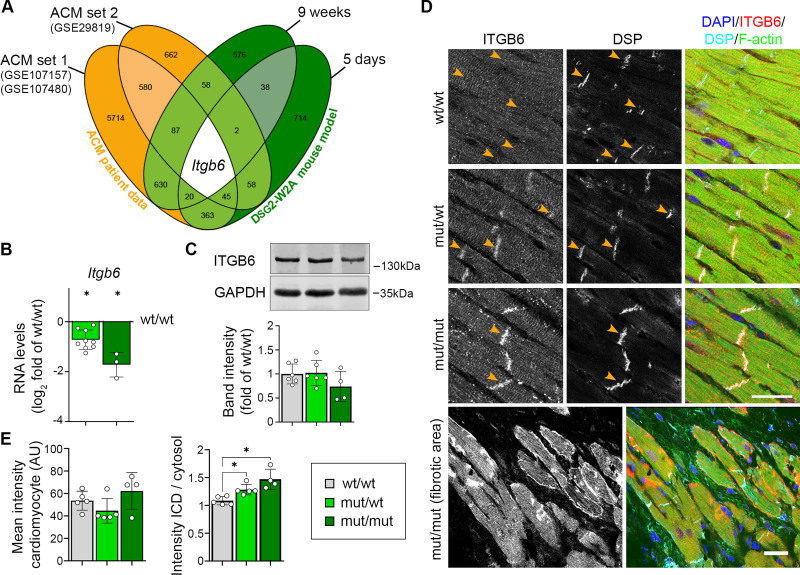
**Integrin-β6 is deregulated in DSG2 (desmoglein-2)–W2A mutants. A**, Venn diagram of significantly altered genes from indicated arrhythmogenic cardiomyopathy (ACM) patient data sets (ACM versus healthy control) and DSG2-W2A mice at the age of 5 days and 9 weeks (mut/mut versus wt/wt) highlighting integrin-β6 (*Itgb6*) as only overlapping gene with same direction of expression in all data sets. Numbers indicate the amount of overlapping genes for the respective overlays. **B**, RNA expression of *Itgb6* analyzed by means of reverse transcription quantitative polymerase chain reaction in adult DSG2-W2A mouse hearts. *P<0.05, unpaired Student *t* test versus wt/wt. *Gapdh* served as reference gene. **C**, Representative Western blot and respective analysis of band intensity of integrin-β6 (ITGB6) in DSG2-W2A hearts. GAPDH served as loading control. 1-way analysis of variance, Dunnett post hoc test. **D** and **E**, Immunostaining of ITGB6 (red in overlay) in DSG2-W2A hearts with corresponding analysis of staining intensity in cardiomyocytes in total and ratio of staining intensity at the intercalated disc (ICD) area (orange arrowheads) versus cardiomyocytes’ cytosolic area. DSP (desmoplakin; cyan) marks ICDs, DAPI (blue) nuclei and F-actin (green) the sarcomere system. Lower row shows an overview image of a fibrotic area in mut/mut hearts. Scale bars, 25 µm. *P<0.05; left graph: Kruskal-Wallis test with Dunn post hoc test; right graph: 1-way analysis of variance, Dunnett post hoc test. Box with color indications of respective groups applies to the entire figure.

To identify common genes deregulated during ACM pathogenesis, we compared the differentially expressed genes from patient data with the results from mutant mice. Here, we included data from 5-day-old mice to identify changes already present before onset of secondary effects attributable to fibrosis. Integrin-β6 (*Itgb6*) was the only transcript consistently deregulated in all data sets (Figure 3A). Its common downregulation in patients with ACM and mutant mice on RNA levels was further confirmed for heterozygous DSG2-W2A mutants compared with WT (Figure 3B). However, protein levels were unaltered in mutant heart samples (Figure 3C). Immunostaining revealed that, in mutant hearts, ITGB6, which mainly localized to a compartment suggestive for the transverse tubules and costameres in wt/wt animals, was enriched at the ICD (Figure 3D and E). Together, these data demonstrate altered ITGB6 mRNA expression as a common feature in ACM patient and DSG2-W2A hearts and an enrichment of the protein at the ICD in mutant murine samples.

### DSG2-W2A Mutant Hearts Present With Structurally Impaired ICDs

**Figure 4.**
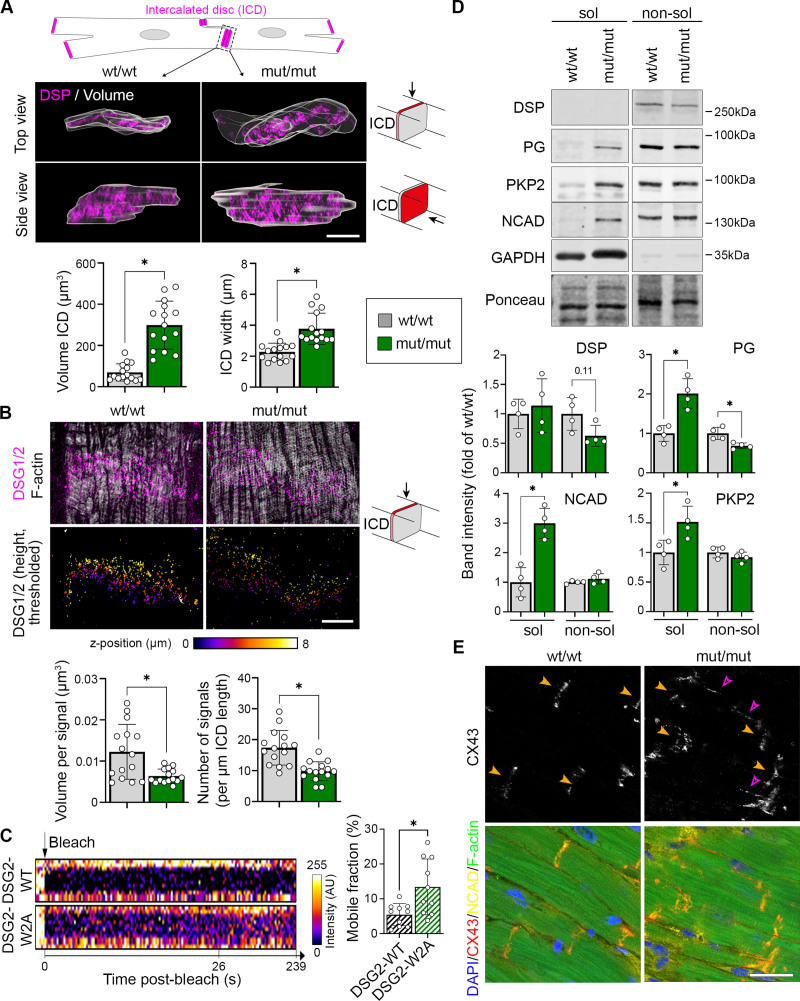
**Disrupted ICD structure in DSG2 (desmoglein-2)–W2A mutant mice. A**, Representative z-stack reconstruction of segmented intercalated discs (ICDs) in top and side view acquired with structured illumination microscopy. Overlay of analyzed ICD volume is shown in gray. ICDs are marked by DSP (desmoplakin; magenta). Scheme on top presents segmented area and pictograms on the right display the respective angle of view. Corresponding analysis of ICD volume and width of ICD between adjacent cardiomyocytes below. Scale bar, 5 µm. *P<0.05, Mann-Whitney test. Each dot represents the value of 1 ICD from 4 mice per genotype. **B**, Representative images of DSG2 (magenta) and filamentary actin (f-actin; white) z-stacks acquired by structured illumination microscopy and presented as maximum intensity projection. Lower row shows color-coded height projection of DSG2 signals in z-stack after signal thresholding as performed for analysis. Related analysis of DSG2 signal volume and number per ICD length is shown below. Pictograms on the right display the respective angle of view. Scale bar, 5 µm. *P<0.05, unpaired Student *t* test, with Welch correction. Each dot represents the value of 1 ICD from in total 3 mice per genotype. **C**, Fluorescence recovery after photobleaching analysis of DSG2–wild type (WT) and DSG2-W2A-mGFP fusion proteins at the cell–cell junction of neonatal cardiomyocytes with representative intensity kymographs of bleached areas on the left. Time point 0 = bleach as indicated by the black arrow. Analysis of the mobile fraction of the indicated mGFP-fusion proteins is shown on the right. *P<0.05, unpaired Student *t* test, with Welch correction. Each dot represents the mean value of 1 heart from in total 3 isolations. **D**, Representative triton-X-100 assay immunoblot with separation of a soluble (sol), noncytoskeletal bound protein fraction from a nonsoluble (non-sol), cytoskeletal-anchored fraction and corresponding analysis shown below. PG (plakoglobin), PKP2 (plakophilin-2), and N-cadherin (NCAD) were analyzed. Intensity of proteins was normalized to the total amount of protein detected by ponceau staining. GAPDH and DSP served as separation control. *P<0.05 or as indicated, unpaired Student *t* test (PG, NCAD, PKP2) or Mann-Whitney test (DSP). Each dot represents the result from 1 mouse. **E**, Immunostaining of connexin-43 (CX43; red in overlay) in DSG2-W2A hearts. NCAD (yellow) marks ICDs, DAPI (blue) nuclei, and F-actin (green) the sarcomere system. Orange arrowheads mark ICD, pink arrowheads highlight lateralization of CX43 staining. Scale bars, 25 µm. Images representative for 5 mice per genotype. Box with color indications of respective groups applies to the entire figure.

This result together with the transmission electron microscopy data (Figure 1K) suggest structural changes of the ICD in mutant hearts, which we addressed further. Using DSP as marker, we reconstructed the outlines of ICDs in wt/wt and mut/mut hearts from 3D stacks captured with structured illumination microscopy. This analysis yielded an increase in ICD volume, in particular attributable to an enhanced width between 2 adjacent cells (Figure 4A). The majority of desmosomal molecules as well as N-cadherin were regularly distributed at mutant ICDs (Figure S5A and S5B). This was corroborated by RNA sequencing data from 5-day-old and 9-week-old animals, which showed no consistent changes of desmosomal, adherens, gap, or tight junction molecules (Figure S5C through S5E). The only adhesion molecule reduced globally was DSG2 (Figure S5A and S5B). Although DSG2 was still present at the ICD, detailed analysis by structured illumination microscopy revealed reduced number and volume of DSG2 signals (Figure 4B). Moreover, fluorescence recovery after photobleaching experiments in neonatal murine cardiomyocytes transduced with DSG2-WT-mGFP or DSG2-W2A-mGFP showed a higher mobility of the mutant protein at cardiomyocyte junctions (Figure 4C), indicating reduced membrane stability of DSG2-W2A. To further investigate the possibility that the stability and integrity of ICD molecules is compromised, we performed Triton-X-100 separation assays for proteins with unaltered ICD localization in mutant hearts. Cytosolic and unanchored membrane proteins are found in the Triton-X-100 soluble fraction, whereas cytoskeleton-bound molecules are mainly detectable in the nonsoluble fraction. Both desmosomal molecules (PG, PKP2) as well as N-cadherin showed increased levels in the soluble pool, indicating reduced cytoskeletal anchorage and impaired ICD integrity in mutants (Figure 4D). Moreover, the gap junction molecule connexin-43, which is required for electrical coupling of cardiomyocytes, was delocalized to the lateral membrane (Figure 4E), which was described as a feature of disrupted ICDs.^8^

These data together with the transmission electron microscopy results demonstrate structurally severely altered ICDs in response to the DSG2-W2A mutation and suggest a molecular basis leading to the functional alterations detectable by echocardiography and ECG.

### Integrin-αV/β6 Is Activated in DSG2-W2A Mutant Hearts

**Figure 5.**
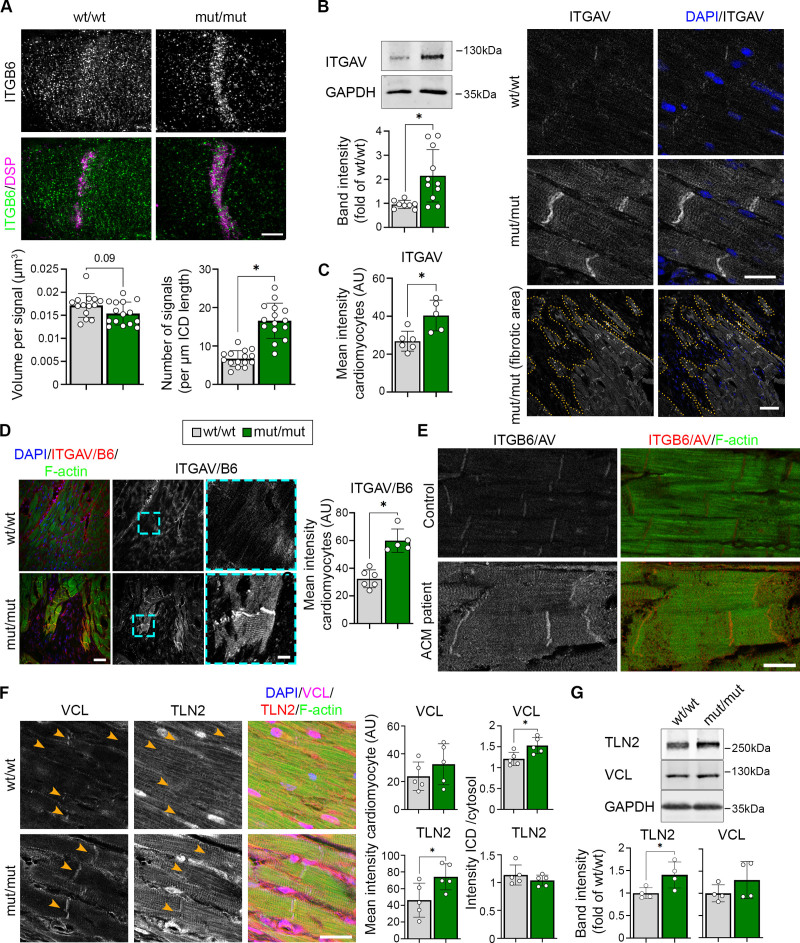
**Increased activation of ITGB6/AV at ICDs of DSG2 (desmoglein-2)–W2A mutant mice. A**, Representative intercalated disc (ICD) reconstruction from z-stacks of integrin-β6 (ITGB6; green) and DSP (magenta) immunostaining acquired by structured illumination microscopy and presented as maximum intensity projection. Related analysis of ITGB6 signal volume and number per ICD length is shown below. Scale bar, 5 µm. *P<0.05 or as indicated, unpaired Student *t* test with Welch correction (left graph) and Mann-Whitney test (right graph). Each dot represents the value of 1 ICD from in total 4 mice per genotype. **B**, Representative Western blot and analysis of band intensity of integrin-αV (ITGAV) in DSG2-W2A hearts. GAPDH served as loading control. *P<0.05, unpaired Student *t* test with Welch correction. **C**, Immunostaining of ITGAV in DSG2-W2A hearts on the right with respective analysis on the left. DAPI (blue) marks nuclei. Lower row shows an overview image of a fibrotic area in mut/mut hearts. Dotted orange line marks the edge of fibrotic area. Scale bars: upper rows, 20 µm; lower row, 50 µm. *P<0.05, unpaired Student *t* test. **D**, Immunostaining of ITGAV/B6 heterodimer in DSG2-W2A mutant hearts with respective analysis of staining intensity on the right. Cyan rectangle marks zoomed area on the right. Scale bars: overview, 50 µm; insert, 10 µm. *P<0.05, unpaired Student *t* test. **E**, Representative immunostaining images of ITGAV/B6 heterodimer staining (red) in a patient with arrhythmogenic cardiomyopathy (ACM; DSP-E952X, heterozygous) and healthy control sample. F-actin (green) stains the sarcomere system. For the patient with ACM, 4 different tissue samples were analyzed and compared with 2 tissue samples from 2 healthy controls. Scale bar, 20 µm. **F**, Immunostaining of VCL (vinculin; magenta) and TLN2 (talin-2; red) in DSG2-W2A hearts on the right with respective analysis of the mean staining intensity in cardiomyocytes and ratio of the staining intensity at the ICD versus cytosolic area on the left. DAPI (blue) marks nuclei. F-actin (green) stains the sarcomere system. Scale bar, 25 µm; *P<0.05, unpaired Student *t* test. **G**, Representative Western blot and analysis of band intensity of VCL and TLN2 in DSG2-W2A hearts. GAPDH served as loading control. *P<0.05, unpaired Student *t* test for TLN2, Mann-Whitney test for VCL. Box with color indications of respective groups applies to the entire figure.

In contrast to DSG2, we detected ITGB6 to be increased at the ICD (Figure 3D and 3E), which was confirmed by analysis of structured illumination microscopy images (Figure 5A). ITGB6 needs to heterodimerize with integrin-αV (ITGAV) in order to be activated and bind to the extracellular matrix.^16–18^ Thus, we analyzed the expression of the counterpart ITGAV, which was significantly upregulated on protein level and increased at the ICD and costameres of mutant hearts (Figure 5B and 5C). In contrast, the localization and intensity of integrin-β1, as classical representative of the integrin group, was not altered (Figure S6). Staining with an antibody specifically recognizing the heterodimer of ITGAV/B6 revealed increased amount of dimer formation at the ICD and costameres in mutant mice (Figure 5D). Moreover, increased ITGAV/B6 staining intensity was also detectable in an ACM patient sample with DSP mutation (Figure 5E). To stabilize active integrins, recruitment of the cytoskeletal adapters talin and vinculin is required.^19^ Accordingly, the expression of talin-2 was increased in DSG2-W2A mut/mut myocardium, with vinculin being enriched at the ICD (Figure 5F and 5G). This effect is similar to what was shown before in response to increased intracellular traction forces on junctions.^20^ Thus, these data suggest that in response to a dysfunctional, nonadhesive ICD with impaired cytoskeletal anchorage, ITGAV/B6 dimers are recruited and activated at the compromised ICD as well as at the costamere region.

### TGF-β Signaling Is Upregulated in DSG2-W2A Mutant Hearts

**Figure 6.**
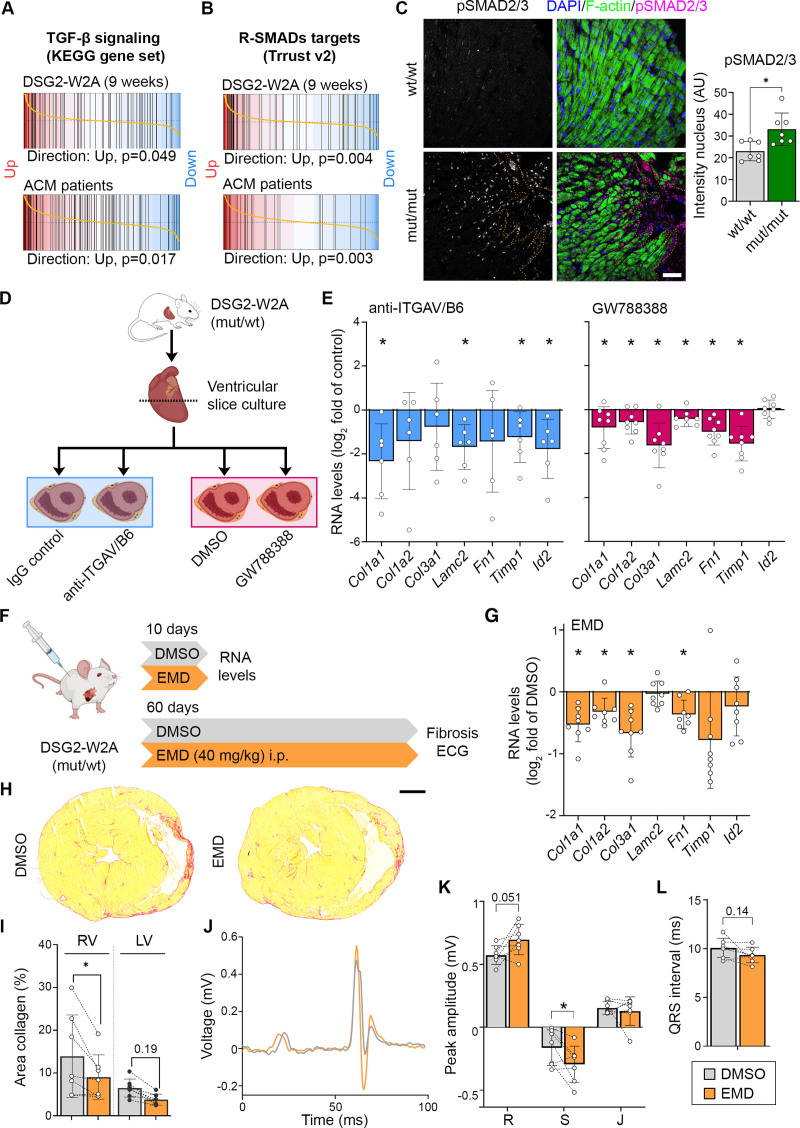
**Elevated transforming growth factor–β signaling in DSG2 (desmoglein-2)–W2A hearts as result of ITGAV/B6 activity.** Barcode plots of gene set enrichment analysis of (**A**) the KEGG_TGF_BETA_SIGNALING_PATHWAY data set (systematic name: M2642)^41^ and (**B**) genes directly regulated by receptor-regulated SMADs (R-SMADs; includes SMAD1/2/3/5/9) as published in the TRRUST database^21^ in 9-week-old DSG2-W2A (mut/mut vs wt/wt) or arrhythmogenic cardiomyopathy (ACM) patient data set 1 (ACM versus healthy control; GEO: GSE107157/GSE107480). Indicated *P* values are calculated by function cameraPR of the R package limma. **C**, Immunostaining of phosphorylated SMAD2/3 (magenta, S465/S467 or S423/S425, respectively) in sections of DSG2-W2A hearts and related analysis of nuclear staining intensity. Nuclei are stained with DAPI (blue), cardiomyocytes are marked with f-actin (green). The dotted orange line marks the edge of the fibrotic area. Scale bar, 50 µm. *P<0.05, unpaired Student *t* test. **D**, Schematic of experimental setup for integrin-αVβ6 (ITGAV/B6) blocking experiments in cardiac slice culture with related results in **E**. Icons are derived from BioRender. **E**, Reverse transcription quantitative polymerase chain reaction analysis of expression of genes downstream of transforming growth factor–β (TGF-β) signaling in cardiac slice cultures treated with inhibiting anti-ITGAV/B6 (1:15) or 10 µmol/L GW788388, an inhibitor of TGF-β receptor I, for 24 hours. *P< 0.05, paired Student *t* test versus indicated control condition. **F**, Schematic of experimental setup for in vivo ITGAV/B6 blocking experiments by injection of 40 mg/kg EMD527040 (EMD) intraperitoneally daily. DMSO was applied as vehicle control. Icons are derived from BioRender. **G**, Reverse transcription quantitative polymerase chain reaction expression analysis of genes downstream of TGF-β signaling in hearts of mice treated with EMD or respective amount of DMSO for 10 days. *P<0.05, unpaired Student *t* test versus DMSO. **H** and **I**, Cardiac fibrosis detected by picrosirius red collagen staining with representative images and corresponding analysis of the area of collagen in the right ventricle (RV) and left ventricle (LV). Lines indicate littermates. Each dot represents 1 animal. *P<0.05 or as indicated, grouped 2-way repeated-measures analysis of variance with LV/RV and experimental pairs matched, Sidak post hoc test. Scale bar, 1 mm. **J** through **L**, ECG recoded in lead II with representative curves shown in J. Corresponding analysis of R, S, and J peak amplitude and QRS interval, *P<0.05 or as indicated. Paired Student *t* test. Lines indicate littermates. Each dot represents 1 animal.

ITGAV/B6 dimers have the ability to activate the profibrotic cytokine transforming growth factor–β (TGF-β) by binding and removal of the latency-associated peptide.^17^ Gene set enrichment analyses revealed an upregulation of genes associated with TGF-β signaling in both 9-week-old mutants as well as patients with ACM (Figure 6A). Moreover, direct targets of receptor-regulated SMADs involved in TGF-β signaling^21^ were upregulated in DSG2-W2A mutant hearts (Figure 6B). Accordingly, increased amounts of nuclear SMAD2/3 phosphorylated at the activation sites S465/S467 or S423/S425, respectively, were detected in these hearts (Figure 6C). Increased levels of pSMAD2/3 were found not only in fibroblasts but also in cardiomyocytes, mainly adjacent to fibrotic areas. These data indicate upregulation of the profibrotic TGF-β pathway and its downstream targets, which include extracellular matrix proteins such as collagens, laminin, or fibronectin.

### Fibrosis in DSG2-W2A Mutant Animals Is Reduced by Inhibition of ITGAV/B6-Dependent Release of TGF-β

On the basis of the known profibrotic role of TGF-β in cardiomyopathies^2^ and the established function of ITGAV/B6 dimers to activate TGF-β,^17^ we investigated whether inhibition of ITGAV/B6 was efficient to reduce fibrosis in DSG2-W2A mice. Cardiac slice cultures were generated from the ventricles of heterozygous mice at the age of 40 to 50 weeks and treated for 24 hours with anti-ITGAV/B6, which was shown to neutralize the function of the dimer.^22^ In comparison, slices were treated with the TGF-β receptor I inhibitor GW788388 to directly block TGF-β signaling (Figure 6D). Significant downregulation in the expression of the profibrotic molecules collagen-I type-α1 (*Col1a1*), laminin subunit-γ2 (*Lamc2*), metalloproteinase inhibitor-1 (*Timp1*), and ID-2 (*Id2*) was induced in the mutant mice in response to inhibition of ITGAV/B6 (Figure 6E). Other extracellular matrix proteins such as fibronectin (*Fn1*) or collagens (*Col3a1*, *Col1a2*) showed the same tendency. All of these fibrotic markers were significantly upregulated in adult mutant hearts as detected by RNA sequencing (Figure 6A and 6B). Direct inhibition of TGF-β led to a similar downregulation of these markers as the blocking antibody.

To investigate the in vivo relevance of this pathway, we applied the small molecule EMD527040 (EMD), which is an established inhibitor of ITGAV/B6-dependent TGF-β release, to the DSG2-W2A mouse model.^23^ For these experiments, we chose wt/mut mice because they reflect the heterozygosity most commonly found in patients with ACM and develop the phenotype during adulthood, when compound application is feasible. Littermates between the ages of 28 and 38 weeks were sex-matched and injected intraperitoneally daily for 10 days with EMD or DMSO control (Figure 6F). In this short-time approach, myocardial samples of EMD-treated animals showed a similar reduction of profibrotic markers as reported for slice cultures (Figure 6G). Having established an effective dose, we performed in vivo studies for a duration of 60 days during the time period when RV fibrosis establishes. At the end of the treatment period, ECG and histologic analysis were performed. EMD-treated mice demonstrated reduced RV fibrosis (Figure 6H and 6I). Moreover, EMD animals showed a mitigation of ECG abnormalities, with significantly higher S amplitude and trends towards an increased R amplitude and shorter QRS interval compared with the vehicle-treated control.

These experiments link ITGAV/B6 dimerization in response to abrogated DSG2 binding and ICD dysfunction to TGF-β–dependent fibrosis generation. The pilot inhibitor studies suggest ITGAV/B6 as a promising treatment target to ameliorate the ACM phenotype.

## Discussion

**Figure 7.**
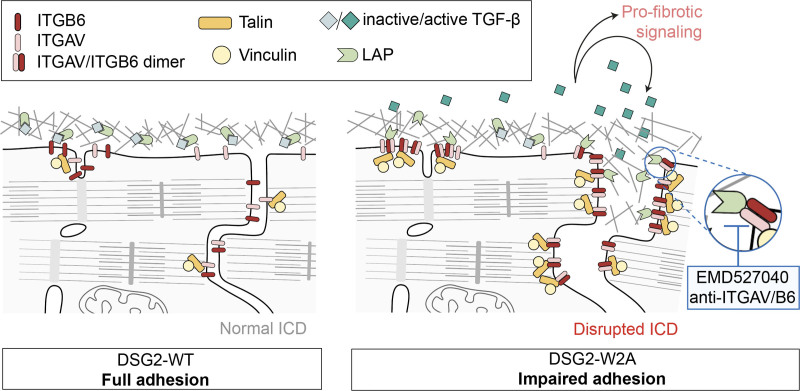
**Schematic conclusion of data.** DSG2 (desmoglein-2) –W2A mutation with loss of desmosomal adhesion leads to impaired intercalated disc (ICD) structure with deregulation of integrin-β6 (ITGB6) and enhanced heterodimerization with integrin-αV (ITGAV). The dimer efficiently binds to the extracellular matrix and activates transforming growth factor–β (TGF-β) by removal of the latency-associated peptide (LAP). Active TGF-β can then induce profibrotic downstream signaling by means of SMAD molecules. In our experiments, this cascade was blocked by different approaches to inhibit ITGAV/B6.

In this study, we generated a knock-in mouse model with defective binding function of the adhesion molecule DSG2, which demonstrated that impaired desmosomal adhesion is sufficient to induce a phenotype mimicking the characteristics of ACM. Our data suggest a cascade of defective desmosomal adhesion, disrupted ICD structure, and subsequent activation of ITGAV/B6 with profibrotic TGF-β signaling as important underlying mechanisms leading to this phenotype (Figure 7). Moreover, our pilot study indicates a beneficial effect of ITGAV/B6 inhibition by EMD treatment with regard to fibrosis and several ECG parameters, suggesting that this pathway can be targeted successfully by drug treatment.

### Defective Desmosomal Adhesion by DSG2-W2A Mutation Is Inducing an ACM Phenotype

Because of mutations mainly affecting desmosomal genes and evidence of disrupted ICDs, the hypothesis of dysfunctional desmosomes with loss of cell–cell adhesion as a central pathologic step was adopted in the field.^2^ However, experimental data on this topic are contradictory.^6–8^ We show that W2 of DSG2 is central for binding and intercellular adhesion in vitro and in vivo. Disrupted desmosomal adhesion is sufficient to induce an ACM phenotype fulfilling the Padua criteria,^13^ including ventricular fibrosis, depolarization and repolarization abnormalities, arrhythmia, and impaired ventricular function, which are used to establish the diagnosis. In line with this, 3 mutations were described in patients with ACM directly affecting the DSG2 binding mechanism by exchange of the tryptophan with a serine, leucine, or arginine (ClinVar data bank: VCV000199796, VCV000044282, VCV000420241).^10^ This indicates a disruption of the tryptophan swap independent from the substituting amino acid and supports defective desmosomal adhesion as important factor in ACM.^2,24^ Moreover, different published DSG2 mutant mouse models show a phenotype resembling the features of DSG2-W2A mutants. These include a DSG2 mutant in which parts of the 2 outermost DSG2 extracellular domains were deleted (but leaving W2A intact),^25^ 2 mouse lines with loss of DSG2,^25,26^ and mice overexpressing a patient mutation (DSG2-N271S),^27^ which also showed that ICD structural aberrations precede functional abnormalities and fibrosis generation. These data fuel the hypothesis that disruption and rearrangement of the ICD in response to impaired adhesion between cardiomyocytes is a crucial initial step, at least in case of mutations in desmosomal molecules. Other mechanisms described in patients or translational models, such as aberrant WNT or Hippo/YAP signaling, or immune cell infiltrations,^2,28^ may be secondary responses and in part even represent adaptive attempts to rescue the functional consequences of such severe structural aberrations.

### DSG2-W2A Mice as Model to Study ACM

Our results suggest the DSG2-W2A model as a valuable tool to study ACM mechanisms. Within the limitations of a murine model, it reproduces a majority of features found in patients with ACM (Table S2). In homozygous animals, both ventricles are affected from early on in life; heterozygous animals develop a milder phenotype, with fibrosis occurring only in the RV. Whether this milder phenotype would extend to the LV over time or whether a gene dose effect underlies the structural differences is unclear. LV and RV remodel differently in response to loading and injury^29^ and it is possible that the RV is less able to compensate a partial reduction of cardiomyocyte cohesion, in contrast to the LV. A fraction of mut/wt animals did not develop the phenotype at all within the observation period of up to 80 weeks. Thus, the model might help clarify why patients with the same mutation (ie, in the same family) develop the disease with variable penetrance.^1^ Similar to patients,^1^ male mutant mice appear to experience more arrhythmia and sudden death than do female mice.

We can interpret from the combined histology, echocardiography, and ECG data that the changes in heart function are secondary to fibrosis generation. This is indicated by the notion that functional measures (eg, ejection fraction, QRS interval) worsened over time in homozygous mutants, whereas no increase was detectable for fibrosis. Moreover, functional changes in mut/wt animals were only observed when fibrosis was present. This is in line with a patient cohort with DSP mutations, in which LV fibrosis preceded systolic dysfunction.^30^ A different study in DSG2-N271S-expressing ex vivo paced hearts demonstrated conduction velocity impairments before onset of fibrosis.^27^ More longitudinal studies using different mutational backgrounds and sensitive detection methods (eg, cardiac magnetic resonance imaging and ECG under exercise) are necessary to define the role of fibrosis as cause or consequence of functional changes.

As with all murine models, the DSG2-W2A line has limitations in its ability to fully recapitulate the human disease phenotype. Mutant mice do not show pronounced replacement with adipose tissue, which is a common characteristic in patient heart samples, but in general is not fully modeled in mice.^24^ Moreover, at least in ECGs under anesthesia, not all mutant animals develop arrhythmia, even if fibrosis is present. However, in contrast to most other ACM models, this knock-in model has the advantage of a single amino acid exchange under the endogenous promotor, thus reducing the possibility of secondary unwanted effects attributable to complete protein absence or overexpression. As in patients, the mutation is present in all cell types and not limited to cardiomyocytes.^2,24^

### DSG2-W2A Leads to Dysfunctional ICDs and ITGAV/B6 Rearrangement

DSG2-W2A led to a severely altered ICD structure consistent with impaired cytoskeletal attachment and ruptured junctions. Whereas these changes provide explanations for compromised intercellular adhesion, we also noted alterations in cell–matrix protein distribution. RNA sequencing before and after onset of the disease phenotype and a comparison with ACM patient datasets identified ITGB6 as commonly deregulated in patients with ACM and DSG2-W2A mice. Although we noted reduced mRNA levels in mutant hearts, the overall protein content was unaltered. This suggests pronounced posttranslational regulation of ITGB6, which was already demonstrated in skeletal muscle.^31^ In mutant hearts, ITGB6 was increased at the ICD together with elevated levels of ITGAV and increased heterodimerization of both molecules. By mechanical force exerted through ITGAV/B6, TGF-β is detached from the latency-associated peptide and can induce signaling by means of TGF-β receptors.^17,32^ In line with higher intracellular forces, talin-2 and vinculin were increased at ICDs, as binding of these molecules activates and stabilizes integrins. A mutual regulation of both adhesive compartments is well-established. As an example, upon loss of N-cadherin, integrins are activated and induce fibronectin deposition.^33^ A similar mechanism is conceivable in DSG2-W2A hearts, either by loss of DSG2 or because of reduced N-cadherin anchorage. So far, only limited data are available on the role of integrins in ACM. A recent study showed downregulation of integrin-β1D leading to ventricular arrhythmia.^34^ Furthermore, knockdown of PKP2 in HL-1 cardiomyocytes was described to deregulate focal adhesions, including integrin-α1.^35^

### Activation of TGF-β Signaling by ITGAV/B6 as Potential Therapeutic Target in ACM

The ITGAV/B6 heterodimer is described as one of the major activators of latent TGF-β1 and TGF-β3.^17^ Although TGF-β signaling is known as general driver of cardiac fibrosis^36^ and more specifically was implicated in ACM,^2^ to our knowledge no data are available on the role of ITGAV/B6 and their regulation of TGF-β signaling in cardiac fibrosis. Uncovering this pathway is of high interest as it offers the possibility to target TGF-β with reduced risk of severe side effects occurring in response to direct inhibition.^37^ We demonstrate a reduction of profibrotic gene expression under ACM conditions in response to ITGAV/B6 inhibition and our pilot study data suggest that a small molecule blocking ITGAV/B6-dependent TGF-β release diminished the generation of fibrosis. Similar approaches using this small molecule or neutralizing antibodies were shown to be protective in murine models of lung, liver, and biliary fibrosis.^23,38,39^ However, because of the heterogeneity of the phenotype in heterozygous animals, more detailed studies with larger sample sizes are required to further substantiate this finding.

Whether fibrosis generation is a contributor to the disease or a protective mechanism to ensure integrity of the heart under conditions of compromised adhesion is unclear. In case of the latter, pharmacologic inhibition of fibrosis generation might be detrimental. Nevertheless, fibrosis is a driver of arrhythmia generation,^40^ which is also supported by our results that ECG abnormalities were reduced by inhibition of fibrosis. Careful studies using different therapeutic approaches to reduce fibrosis in appropriate model organisms need to address this aspect in detail.

In conclusion, we established a new mouse model phenocopying many aspects of ACM, uncovered a novel pathway of fibrosis induction, and identified an approach to target this mechanism with future implication as a potential therapeutic option.

## Article Information

### Acknowledgments

The authors thank Alain Brühlhart and the team from the Animal Facility; Dr Cinzia Tiberi (Center for Cellular Imaging and NanoAnalytics); Dr Alexia Loynton-Ferrand (Imaging Core Facility); Dr Diego Calabrese (Histology Core Facility); and Drs Mike Abanto and Beat Erne, P. Lorentz, and E. Bartoszek (Microscopy Core Facility), University of Basel, Switzerland; and Dr Christian Beisel and Philippe Demougin, Genomics Facility Basel (D-BSSE, ETH Zürich, and University of Basel), for support; Nicolas Schlegel (Department of Surgery, University Hospital Würzburg, Germany) for providing CaCo2 cells; Nikola Golenhofen (Institute of Anatomy, University of Ulm, Germany) for providing the Fc-His-pEGFP-N3 plasmid; and Anja Fuchs for technical assistance. Calculations were performed at the sciCORE scientific computing center at University of Basel.

### Sources of Funding

This project was supported by the German Research Council (SP1300-3/1 to Dr Spindler), the Swiss National Science Foundation (197764 to Dr Spindler), the Swiss Heart Foundation (FF21098 to Drs Schinner and Spindler), the Research Fund Junior Researchers, University of Basel (3BM1079 to Dr Schinner), and the Theiler-Haag Foundation (Dr Spindler). Dr Sheikh is supported by grants from the National Institutes of Health (grant HL142251) and Department of Defense (grant W81XWH1810380).

### Disclosures

Dr Sheikh is a cofounder and equity shareholder of Papillon Therapeutics Inc and is a consultant and equity shareholder of LEXEO Therapeutics Inc. The other authors have nothing to disclose.

### Supplemental Material

Expanded Methods

Tables S1–S3

Figures S1–S6

References 42–48

## Supplementary Material

## References

[1] Corrado D Link MS Calkins H. Arrhythmogenic right ventricular cardiomyopathy. N Engl J Med. 2017;376:1489–1490. doi: 10.1056/NEJMc1701400 10.1056/NEJMc1701400 28402769

[2] Austin KM Trembley MA Chandler SF Sanders SP Saffitz JE Abrams DJ Pu WT. Molecular mechanisms of arrhythmogenic cardiomyopathy. Nat Rev Cardiol. 2019;16:519–537. doi: 10.1038/s41569-019-0200-7 31028357 10.1038/s41569-019-0200-7 PMC6871180

[3] Delva E Tucker DK Kowalczyk AP. The desmosome. Cold Spring Harb Perspect Biol. 2009;1:a002543. doi: 10.1101/cshperspect.a002543 20066089 10.1101/cshperspect.a002543 PMC2742091

[4] Delmar M McKenna WJ. The cardiac desmosome and arrhythmogenic cardiomyopathies: from gene to disease. Circ Res. 2010;107:700–714. doi: 10.1161/CIRCRESAHA.110.223412 20847325 10.1161/CIRCRESAHA.110.223412

[5] Basso C Czarnowska E Della Barbera M Bauce B Beffagna G Wlodarska EK Pilichou K Ramondo A Lorenzon A Wozniek O. Ultrastructural evidence of intercalated disc remodelling in arrhythmogenic right ventricular cardiomyopathy: an electron microscopy investigation on endomyocardial biopsies. Eur Heart J. 2006;27:1847–1854. doi: 10.1093/eurheartj/ehl095 16774985 10.1093/eurheartj/ehl095

[6] Hariharan V Asimaki A Michaelson JE Plovie E MacRae CA Saffitz JE Huang H. Arrhythmogenic right ventricular cardiomyopathy mutations alter shear response without changes in cell–cell adhesion. Cardiovasc Res. 2014;104:280–289. doi: 10.1093/cvr/cvu212 25253076 10.1093/cvr/cvu212 PMC4296114

[7] Huang H Asimaki A Lo D McKenna W Saffitz J. Disparate effects of different mutations in plakoglobin on cell mechanical behavior. Cell Motil Cytoskeleton. 2008;65:964–978. doi: 10.1002/cm.20319 18937352 10.1002/cm.20319 PMC2650236

[8] Schlipp A Schinner C Spindler V Vielmuth F Gehmlich K Syrris P McKenna WJ Dendorfer A Hartlieb E Waschke J. Desmoglein-2 interaction is crucial for cardiomyocyte cohesion and function. Cardiovasc Res. 2014;104:245–257. doi: 10.1093/cvr/cvu206 25213555 10.1093/cvr/cvu206

[9] Harrison OJ Brasch J Lasso G Katsamba PS Ahlsen G Honig B Shapiro L. Structural basis of adhesive binding by desmocollins and desmogleins. Proc Natl Acad Sci USA. 2016;113:7160–7165. doi: 10.1073/pnas.1606272113 27298358 10.1073/pnas.1606272113 PMC4932976

[10] NM_001943.5(DSG2). In: ClinVar database. Accessed June 1, 2022. https://www.ncbi.nlm.nih.gov/clinvar/?gr=1&term=NM_001943.5

[11] Bell GI. Models for the specific adhesion of cells to cells. Science. 1978;200:618–627. doi: 10.1126/science.347575 347575 10.1126/science.347575

[12] Meir M Burkard N Ungewiss H Diefenbacher M Flemming S Kannapin F Germer CT Schweinlin M Metzger M Waschke J. Neurotrophic factor GDNF regulates intestinal barrier function in inflammatory bowel disease. J Clin Invest. 2019;129:2824–2840. doi: 10.1172/JCI120261 31205031 10.1172/JCI120261 PMC6597228

[13] Corrado D Perazzolo Marra M Zorzi A Beffagna G Cipriani A Lazzari M Migliore F Pilichou K Rampazzo A Rigato I. Diagnosis of arrhythmogenic cardiomyopathy: the Padua criteria. Int J Cardiol. 2020;319:106–114. doi: 10.1016/j.ijcard.2020.06.005 32561223 10.1016/j.ijcard.2020.06.005

[14] Song JS Wang RS Leopold JA Loscalzo J. Network determinants of cardiovascular calcification and repositioned drug treatments. FASEB J. 2020;34:11087–11100. doi: 10.1096/fj.202001062R 32638415 10.1096/fj.202001062R PMC7497212

[15] Gaertner A Schwientek P Ellinghaus P Summer H Golz S Kassner A Schulz U Gummert J Milting H. Myocardial transcriptome analysis of human arrhythmogenic right ventricular cardiomyopathy. Physiol Genomics. 2012;44:99–109. doi: 10.1152/physiolgenomics.00094.2011 22085907 10.1152/physiolgenomics.00094.2011

[16] Margadant C Sonnenberg A. Integrin-TGF-beta crosstalk in fibrosis, cancer and wound healing. EMBO Rep. 2010;11:97–105. doi: 10.1038/embor.2009.276 20075988 10.1038/embor.2009.276 PMC2828749

[17] Munger JS Sheppard D. Cross talk among TGF-beta signaling pathways, integrins, and the extracellular matrix. Cold Spring Harb Perspect Biol. 2011;3:a005017. doi: 10.1101/cshperspect.a005017 21900405 10.1101/cshperspect.a005017 PMC3220354

[18] Koivisto L Bi J Hakkinen L Larjava H. Integrin alphavbeta6: structure, function and role in health and disease. Int J Biochem Cell Biol. 2018;99:186–196. doi: 10.1016/j.biocel.2018.04.013 29678785 10.1016/j.biocel.2018.04.013

[19] Horton ER Humphries JD James J Jones MC Askari JA Humphries MJ. The integrin adhesome network at a glance. J Cell Sci. 2016;129:4159–4163. doi: 10.1242/jcs.192054 27799358 10.1242/jcs.192054 PMC5117201

[20] Merkel CD Li Y Raza Q Stolz DB Kwiatkowski AV. Vinculin anchors contractile actin to the cardiomyocyte adherens junction. Mol Biol Cell. 2019;30:2639–2650. doi: 10.1091/mbc.E19-04-0216 31483697 10.1091/mbc.E19-04-0216 PMC6761764

[21] Han H Cho JW Lee S Yun A Kim H Bae D Yang S Kim CY Lee M Kim E. TRRUST v2: an expanded reference database of human and mouse transcriptional regulatory interactions. Nucleic Acids Res. 2018;46:D380–D386. doi: 10.1093/nar/gkx1013 29087512 10.1093/nar/gkx1013 PMC5753191

[22] Weinreb PH Simon KJ Rayhorn P Yang WJ Leone DR Dolinski BM Pearse BR Yokota Y Kawakatsu H Atakilit A. Function-blocking integrin alphavbeta6 monoclonal antibodies: distinct ligand-mimetic and nonligand-mimetic classes. J Biol Chem. 2004;279:17875–17887. doi: 10.1074/jbc.M312103200 14960589 10.1074/jbc.M312103200

[23] Patsenker E Popov Y Stickel F Jonczyk A Goodman SL Schuppan D. Inhibition of integrin alphavbeta6 on cholangiocytes blocks transforming growth factor-beta activation and retards biliary fibrosis progression. Gastroenterology. 2008;135:660–670. doi: 10.1053/j.gastro.2008.04.009 18538673 10.1053/j.gastro.2008.04.009 PMC3505071

[24] Gerull B Brodehl A. Genetic animal models for arrhythmogenic cardiomyopathy. Front Physiol. 2020;11:624. doi: 10.3389/fphys.2020.00624 32670084 10.3389/fphys.2020.00624 PMC7327121

[25] Kant S Holthofer B Magin TM Krusche CA Leube RE. Desmoglein 2-dependent arrhythmogenic cardiomyopathy is caused by a loss of adhesive function. Circ Cardiovasc Genet. 2015;8:553–563. doi: 10.1161/CIRCGENETICS.114.000974 26085008 10.1161/CIRCGENETICS.114.000974

[26] Chelko SP Asimaki A Andersen P Bedja D Amat-Alarcon N DeMazumder D Jasti R MacRae CA Leber R Kleber AG. Central role for GSK3beta in the pathogenesis of arrhythmogenic cardiomyopathy. JCI Insight. 2016;1: doi: 10.1172/jci.insight.85923 10.1172/jci.insight.85923 PMC4861310 27170944

[27] Rizzo S Lodder EM Verkerk AO Wolswinkel R Beekman L Pilichou K Basso C Remme CA Thiene G Bezzina CR. Intercalated disc abnormalities, reduced Na(+) current density, and conduction slowing in desmoglein-2 mutant mice prior to cardiomyopathic changes. Cardiovasc Res. 2012;95:409–418. doi: 10.1093/cvr/cvs219 22764152 10.1093/cvr/cvs219

[28] Meraviglia V Alcalde M Campuzano O Bellin M. Inflammation in the pathogenesis of arrhythmogenic cardiomyopathy: secondary event or active driver? Front Cardiovasc Med. 2021;8:784715. doi: 10.3389/fcvm.2021.784715 34988129 10.3389/fcvm.2021.784715 PMC8720743

[29] Friedberg MK Redington AN. Right versus left ventricular failure: differences, similarities, and interactions. Circulation. 2014;129:1033–1044. doi: 10.1161/CIRCULATIONAHA.113.001375 24589696 10.1161/CIRCULATIONAHA.113.001375

[30] Smith ED Lakdawala NK Papoutsidakis N Aubert G Mazzanti A McCanta AC Agarwal PP Arscott P Dellefave-Castillo LM Vorovich EE. Desmoplakin cardiomyopathy, a fibrotic and inflammatory form of cardiomyopathy distinct from typical dilated or arrhythmogenic right ventricular cardiomyopathy. Circulation. 2020;141:1872–1884. doi: 10.1161/CIRCULATIONAHA.119.044934 32372669 10.1161/CIRCULATIONAHA.119.044934 PMC7286080

[31] Ducceschi M Clifton LG Stimpson SA Billin AN. Post-transcriptional regulation of ITGB6 protein levels in damaged skeletal muscle. J Mol Histol. 2014;45:329–336. doi: 10.1007/s10735-014-9567-2 24488487 10.1007/s10735-014-9567-2 PMC3983900

[32] Dong X Zhao B Iacob RE Zhu J Koksal AC Lu C Engen JR Springer TA. Force interacts with macromolecular structure in activation of TGF-beta. Nature. 2017;542:55–59. doi: 10.1038/nature21035 28117447 10.1038/nature21035 PMC5586147

[33] Julich D Cobb G Melo AM McMillen P Lawton AK Mochrie SG Rhoades E Holley SA. Cross-scale integrin regulation organizes ECM and tissue topology. Dev Cell. 2015;34:33–44. doi: 10.1016/j.devcel.2015.05.005 26096733 10.1016/j.devcel.2015.05.005 PMC4496283

[34] Wang Y Li C Shi L Chen X Cui C Huang J Chen B Hall DD Pan Z Lu M. Integrin beta1D deficiency-mediated RyR2 dysfunction contributes to catecholamine-sensitive ventricular tachycardia in arrhythmogenic right ventricular cardiomyopathy. Circulation. 2020;141:1477–1493. doi: 10.1161/CIRCULATIONAHA.119.043504 32122157 10.1161/CIRCULATIONAHA.119.043504 PMC7200284

[35] Puzzi L Borin D Gurha P Lombardi R Martinelli V Weiss M Andolfi L Lazzarino M Mestroni L Marian AJ. Knock down of plakophillin 2 dysregulates adhesion pathway through upregulation of miR200b and alters the mechanical properties in cardiac cells. Cells. 2019;8:1639. doi: 10.3390/cells8121639 10.3390/cells8121639 PMC6952926 31847412

[36] Frangogiannis NG. Cardiac fibrosis: cell biological mechanisms, molecular pathways and therapeutic opportunities. Mol Aspects Med. 2019;65:70–99. doi: 10.1016/j.mam.2018.07.001 30056242 10.1016/j.mam.2018.07.001

[37] Kulkarni AB Huh CG Becker D Geiser A Lyght M Flanders KC Roberts AB Sporn MB Ward JM Karlsson S. Transforming growth factor beta 1 null mutation in mice causes excessive inflammatory response and early death. Proc Natl Acad Sci USA. 1993;90:770–774. doi: 10.1073/pnas.90.2.770 8421714 10.1073/pnas.90.2.770 PMC45747

[38] Puthawala K Hadjiangelis N Jacoby SC Bayongan E Zhao Z Yang Z Devitt ML Horan GS Weinreb PH Lukashev ME. Inhibition of integrin alpha(v)beta6, an activator of latent transforming growth factor-beta, prevents radiation-induced lung fibrosis. Am J Respir Crit Care Med. 2008;177:82–90. doi: 10.1164/rccm.200706-806OC 17916808 10.1164/rccm.200706-806OC PMC2176115

[39] John AE Graves RH Pun KT Vitulli G Forty EJ Mercer PF Morrell JL Barrett JW Rogers RF Hafeji M. Translational pharmacology of an inhaled small molecule alphavbeta6 integrin inhibitor for idiopathic pulmonary fibrosis. Nat Commun. 2020;11:4659. doi: 10.1038/s41467-020-18397-6 32938936 10.1038/s41467-020-18397-6 PMC7494911

[40] Maione AS Pilato CA Casella M Gasperetti A Stadiotti I Pompilio G Sommariva E. Fibrosis in arrhythmogenic cardiomyopathy: the phantom thread in the fibro-adipose tissue. Front Physiol. 2020;11:279. doi: 10.3389/fphys.2020.00279 32317983 10.3389/fphys.2020.00279 PMC7147329

[41] Liberzon A Subramanian A Pinchback R Thorvaldsdottir H Tamayo P Mesirov JP. Molecular signatures database (MSigDB) 3.0. Bioinformatics. 2011;27:1739–1740. doi: 10.1093/bioinformatics/btr260 21546393 10.1093/bioinformatics/btr260 PMC3106198

[42] Concordet JP Haeussler M. CRISPOR: intuitive guide selection for CRISPR/Cas9 genome editing experiments and screens. Nucleic Acids Res. 2018;46:W242–W245. doi: 10.1093/nar/gky354 29762716 10.1093/nar/gky354 PMC6030908

[43] Ebner A Wildling L Kamruzzahan AS Rankl C Wruss J Hahn CD Holzl M Zhu R Kienberger F Blaas D. A new, simple method for linking of antibodies to atomic force microscopy tips. Bioconjug Chem. 2007;18:1176–1184. doi: 10.1021/bc070030s 17516625 10.1021/bc070030s

[44] Hutter JL Bechhoefer J. Calibration of atomic-force microscope tips. Rev Sci Instrum. 1993;64:1868–1873. doi: 10.1063/1.1143970

[45] Dobin A Davis CA Schlesinger F Drenkow J Zaleski C Jha S Batut P Chaisson M Gingeras TR. STAR: ultrafast universal RNA-seq aligner. Bioinformatics. 2013;29:15–21. doi: 10.1093/bioinformatics/bts635 23104886 10.1093/bioinformatics/bts635 PMC3530905

[46] Liao Y Smyth GK Shi W. featureCounts: an efficient general purpose program for assigning sequence reads to genomic features. Bioinformatics. 2014;30:923–930. doi: 10.1093/bioinformatics/btt656 24227677 10.1093/bioinformatics/btt656

[47] Chopin M Preston SP Lun ATL Tellier J Smyth GK Pellegrini M Belz GT Corcoran LM Visvader JE Wu L. RUNX2 mediates plasmacytoid dendritic cell egress from the bone marrow and controls viral immunity. Cell Rep. 2016;15:866–878. doi: 10.1016/j.celrep.2016.03.066 27149837 10.1016/j.celrep.2016.03.066

[48] Ehler E Moore-Morris T Lange S. Isolation and culture of neonatal mouse cardiomyocytes. J Vis Exp. 2013;79:50154. doi: 10.3791/50154 10.3791/50154 PMC3857885 24056408

