# Supplementary material for: Defective Desmosomal Adhesion Causes Arrhythmogenic Cardiomyopathy by Involving an Integrin-αVβ6/TGF-β Signaling Cascade
Source: Circulation. 2022 Oct 21;146(21):1610–26. doi: 10.1161/CIRCULATIONAHA.121.057329 (PMC9674449; doi:10.1161/CIRCULATIONAHA.121.057329)
Supplement: Supplementary file 1 [file cir-146-1610-s001.pdf]

SUPPLEMENTAL MATERIAL

Defective Desmosomal Adhesion Causes Arrhythmogenic Cardiomyopathy by involving an Integrin-αVβ6/TGF-β Signaling Cascade

Camilla Schinner MD, Lifen Xu PhD, Henriette Franz PhD, Aude Zimmermann M.sc., Marie-Therès Wanuske M.sc., Maitreyi Rathod PhD, Pauline Hanns PhD, Florian Geier PhD, Pawel Pelczar PhD, Yan Liang PhD, Vera Lorenz M.Sc., Chiara Stüdle PhD, Piotr I Maly PhD, Silke Kauferstein PhD, Britt Maria Beckmann MD, Farah Sheikh PhD, Gabriela M Kuster MD, Volker Spindler MD

1. ***Extended Methods***

DSG2-W2A mouse model

All mouse experiments were carried out according to the protocol approved by the Cantonal Veterinary Office of Basel-Stadt (License number 2973_32878 and 3070_32419). All mice were housed under specific pathogen-free conditions with standard chow and bedding with 12 hours day/night cycle according to institutional guidelines. Animals of both sexes were applied without bias. For inhibitor treatments, all mice were age- and sex-matched and randomly allocated to treatment or control group.

The Dsg2-W>A allele was obtained by Cas9/CRISPR embryo electroporation. The Cas9/CRISPR target sequence tggttcgtcaaaagagggcc(tgg) (PAM sequence in brackets is also the TGG-Trp codon) spanning the mutation site was selected with the help of CRISPOR software (http://crispor.tefor.net/)[42]. ssDNA oligonucleotide 5’gtgataactcaaggtaattgtattaacaggtcttcagccc aagaaatgaaggcaaaccgttccctaagcacactcacttggttcgtcaaaagagggca**<u>gct</u>**atcactgcccctgtgg ctctgcgggagggcgaagacctgtccagaaagaacccgattgccaaggtagcagctacagaagaatgtggcgag ggtgttggc3’ (GCT – Ala codon in bold underlined) was designed to insert the W>A mutation into the Cas9-generated DSB by homologous recombination and at the same time mutate the TGG PAM sequence to GCT. C57BL/6J female mice underwent ovulation induction by i.p. injection of 5 IU equine chorionic gonadotrophin (PMSG; Folligon, InterVet, Vienna, Austria), followed by i.p. injection of 5 IU human chorionic gonadotropin (Pregnyl, Essex Chemie, Lucerne, Switzerland) 48 hours later. For the recovery of embryos, C57BL/6J females were mated with males of the same strain immediately after the administration of human chorionic gonadotropin. Embryos were collected from oviducts 24 hours after the human chorionic gonadotropin injection, and were then freed from any remaining cumulus cells by a 1–2 min treatment of 0.1 %

hyaluronidase (Sigma-Aldrich, St. Louis, MO, ISA) dissolved in M2 medium (Sigma-Aldrich). Prior to electroporation, the zona pellucida was partially removed by brief treatment with acid Tyrode's solution and the embryos were washed and briefly cultured in M16 medium (Sigma-Aldrich) at 37 °C and 5 % CO_2. Electroporation with a mixture of ssDNA oligonucleotide targeting template, 16 µmol/l cr:trcrRNA hybrid targeting Dsg2 and 16 µmol/l Cas9 protein (all reagents from IDT, Coralville, IA, USA) was carried out using 1 mm gap electroporation cuvette and the ECM830 electroporator (BTX Harvard Apparatus, Holliston, MA, USA). Two square 3 ms pulses of 30 V with 100 ms interval were applied. Surviving embryos were washed with M16 medium and transferred immediately into the oviducts of 8–16-weeks-old pseudopregnant Crl:CD1(ICR) females that had been mated with sterile genetically vasectomized males the day before embryo transfer (0.5 dpc). Pregnant females were allowed to deliver and raise their pups until weaning age. In total 150 embryos were electroporated and 147 surviving embryos were transferred into 7 foster mothers. All foster mothers produced live litters with a total of 20 viable F0 pups. One F0 pup carried the desired mutation as confirmed by sequencing. This founder animal was bred to C57BL/6J partner. The mut/wt offspring from this mating was bred to C57BL/6J partners for 2 generations to establish the Dsg2-W2A mouse line.

For genotyping of the DSG2-W2A line, DNA was extracted from biopsies in 25 mmol/l NaOH and 0.2 mmol/l EDTA at 98 °C for 1 hour and neutralized with 40 mmol/l Tris pH 5.5. PCR was performed using GoTaq G2 (M7845, Promega, Madison, WI, USA) according to manufacturer's instructions with the primers Dsg2-W2A for: GAATGTCTCCCCAAAGCTTTGGGTATG and Dsg2-W2A rev: CTGCTACCTTGGCAATCGGGTTC, which span the mutated region. The PCR product was restricted with 66.7 U/ml AluI (R0137, New England Biolabs, Ipswich, MA, USA) in CutSmart buffer (New England Biolabs) overnight at 37 °C. By subsequent

electrophoresis in a 3 % (w/v) agarose gel containing Midori Green Advanced (Nippon Genetics, Düren, Germany) for fluorescence DNA visualization, presence of a Dsg2-WT allele was detectable as 197 bp fragment, while Dsg2-W2A mutant allele was cut into a 109 bp and 72 bp fragment (Figure S1C).

Murine sample collection

For heart dissection, mice were euthanized via i.p. pentobarbital overdose according to guidelines of the Cantonal Veterinary Office of Basel-Stadt and the University of Basel. Hearts were removed by lateral thoracotomy and directly immersed in ice-cold HBSS supplemented with 20 mmol/l 2,3-Butanedione monoxime (BDM, Sigma-Aldrich) unless stated otherwise. Morphology of the hearts was analyzed using a binocular stereo microscope (SZX2, Olympus, Shinjuku, Japan) and documented with a SLR camera (EOS 800D, Canon, Tokyo, Japan). Tissue was processed as described in the respective section.

For dissection of embryos, timed matings were performed and pregnant mice euthanized via i.p. pentobarbital overdose after the respective days. Embryos were dissected from the uterus and placed in HBSS. After image acquisition as described above, a tissue sample for genotyping was collected from the tail and embryos were processed as described in the *Histological staining* section.

In vivo experiments

At begin of the experiments mut/wt mice from the same litter were sex-matched and randomly allocated by stratified randomization to control or treatment group. Within the limits of a pilot experiment, number of animals were estimated with a power calculation. EMD527040 hydrochloride (EMD, N-[1-Oxo-5-(2-pyridinylamino)pentyl]-O-(phenylmethyl)-L-seryl-3-(3,5-dichlorophenyl)-β-alanine hydrochloride, 637.47g/mol, custom made Cat. No. 7508, Tocris, Bristol, UK) was dissolved in DMSO at 60 mg/ml. For i.p. application, EMD was mixed 2:1 with sterile PBS and administered daily at a

concentration of 40 mg/kg body weight for the indicated time. 2:1 DMSO in PBS was administered to the control group. During treatment, physical status of mice was controlled daily. ECG measurements, sample collection and analysis were performed as described in the respective section.

Plasmid generation and cloning

For lentiviral overexpression plasmids, DNA for full length Dsg2-WT and full length Dsg2-W2A mutation, respectively, were amplified from cDNA originating from liver tissue of DSG2-WT and DSG2-W2A mice using AscI-msDsg2-N forward and NotI-GT-msDsg2-C reverse primers. Amplicons were ligated into AscI and NotI digested pLENTI-C-mGFP (#PS100071, OriGene, Rockville, MD, USA) according to standard procedures. To produce the proteins used in the AFM experiments, the signal, pro-peptide, and all extracellular domains of Dsg2-WT and Dsg2-W2A, respectively, were amplified from murine cDNA using AfeI-Kozak-msDsg2-N forward and XhoI-msDsg2-C reverse primers. Amplicons were ligated into AfeI and XhoI digested Fc-His-pEGFP-N3 plasmid containing the Fc domain from human IGHG1 (bases 295-990) (a kind gift of Nikola Golenhofen, Institute of Anatomy and Cell Biology, University of Ulm, Ulm, Germany). For the Fc control construct, the signal peptide of murine interleukin 2 was inserted N-terminally of the human Fc by annealing the oligomers NheI-Kozak-IL2 Signal and XhoI-IL2 Signal and ligating them into the NheI and XhoI digested Fc-His-pEGFP-N3 plasmid.

Primers and oligomers for cloning

AscI-msDsg2-N forward: GTTTGGCGCGCCATGGCGCGGAGCCCGGGT

NotI-GT-msDsg2-C reverse: GTTTGCGGCCGCGTGGAGTAAGAATGTTGCATGGTG

AfeI-Kozak-msDsg2-N forward: GTTTAGCGCTGCCACC ATGGCGCGGAGCCCGGGTGA

XhoI-msDsg2-C reverse: GTTTCTCGAGGGCAGGGCCCAACCCGAC

NheI-Kozak-IL2 Signal: CTAGCCACCATGTACAGGATGCAACTCCTGTCTTGCATTGCACTAAGTCTTGCA CTTGTCACGAATTCGC

XhoI-IL2 Signal: TCGAGCGAATTCGTGACAAGTGCAAGACTTAGTGCAATGCAAGACAGGAGTTGC ATCCTGTACATGGTGG

Cultivation of CaCo2 cells

The human intestinal cell line CaCo2 with WT and DSG2 KO background were kindly provided by Nicolas Schlegel (Department of General, Visceral, Vascular and Pediatric Surgery, University Hospital Würzburg, Würzburg, Germany) and generated as described. [12] CaCo2 cells were maintained in Dulbecco's Modified Eagle Medium (DMEM, D6546, Sigma-Aldrich) supplemented with 10 % foetal bovine serum (S0615, Merck, Darmstadt, Germany), 100 µg/ml penicillin/streptomycin (Applichem, Darmstadt, Germany) and 2 mmol/l L-glutamine (Sigma-Aldrich) at 37 °C, 5 % CO_2 and full humidity. For experiments, cells were seeded on TC-treated plastic cell culture plates, grown to confluency and differentiated for seven days. All cells were quarterly checked for mycoplasma contaminations using PCR and were proven negative. CaCo2 cells were routinely authenticated by Short Tandem Repeat profiling.

Lentivirus generation and transduction

Lentiviral particles were generated according to standard procedures. In brief, HEK293T cells were co-transfected with the packaging vector psPAX2 (#12259, Addgene, Watertown, MA, USA), the envelope vector pMD2.G (#12260, Addgene) and the respective construct plasmid using TurboFect (Thermo Fisher Scientific, Waltham, MA, USA). After 48 hours, virus particle containing supernatant was collected and enriched using LentiConcentrator (OriGene). Cells were transduced with the

respective concentrated virus particles using 10 µg/mL polybrene (Sigma-Aldrich) according to the manufacturer's instructions. After 24 hours, medium was changed and cells cultivated for one week before starting with the respective experiments. Expression of the respective construct was confirmed via Western blot analysis.

Generation and cultivation of murine keratinocytes

For isolation of murine keratinocytes from DSG2-W2A mut/mut and wt/wt mice, the epidermis of neonatal mice was separated from the dermis via incubation in dispase II solution (>2.4 U/ml dispase II, D4693, Sigma-Aldrich, in PBS) with 2x gentamicin/amphotericin B (CELLnTEC, Bern, Switzerland) over night at 4 °C. Keratinocytes were isolated by accutase (A6964, Sigma-Aldrich) for 20 minutes at room temperature and subsequent agitation. Released cells were cultured on collagen I coated plates (50201, IBIDI, Gräfelfing, Germany) in 0.06 mmol/l calcium murine keratinocyte medium (DMEM: Ham's F12 3.5:1.1 mixture, SO-41660, PAN-Biotech, Aidenbach, Germany) supplemented with 10 % calcium-free foetal bovine serum (S0615, Merck), 2 mmol/l stable glutamine (BioConcept, Allschwil, Switzerland), 50µg/ml penicillin/streptomycin (Applichem), 10 ng/ml murine epidermal GF (Invitrogen, Carlsbad, CA), 1 mmol/l sodium pyruvate, 0.18 mmol/l adenine, 120 pmol/l cholera toxin, 5 µg/ml insulin, and 500 ng/ml hydrocortisone (all Sigma-Aldrich). Cells were kept in an incubator at 35 °C with 5 % CO_2 and 100 % humidity, the medium was changed every third day. When reaching confluency, cells were transferred into a new coated culture dish. After around six passages, cells were immortalized and could be expanded and seeded for experiments. 48 hours before experiments were conducted, 1.8 mmol/l calcium was added to the medium to induce cell differentiation. Cells were quarterly checked for mycoplasma infections using PCR and were proven negative.

Cardiac slice culture

Mice were sacrificed by i.p. injection of pentobarbital. The heart was dissected by lateral thoracotomy and placed in ice-cold oxygenated slicing buffer (136 mmol/l NaCl, 5.4 mmol/l KCl, 1 mmol/l $MgHPO_4$, 0.9 mmol/l $CaCl_2$, 30 mmol/l 2,3-Butanedione monoxime, 5 mmol/l HEPES, 10 mmol/l glucose). After removal of both atria, the heart was embedded in 37 °C low melt agarose (Carl Roth, Karlsruhe, Germany) dissolved in slicing buffer without glucose. Using a LeicaVT1200 vibratome (Leica Biosystems, Nussloch, Germany), 300 µm thick tissue sections were cut with 1 mm amplitude and 0.07 mm/s speed. Freshly cut sections were transferred into ice-cold slicing buffer. For incubations, sections were transferred to 0.4 µm polycarbonate membranes cell culture inserts (VWR, Radnor, PA, USA) and incubated in Claycomb medium supplemented with 10 % foetal calf serum, 2 mmol/l L-glutamine, 10U/l : 10µg/ml penicillin and streptomycin (all from Sigma-Aldrich) at 37 °C, 5 % CO2 and treated with either rabbit anti-ITGαV/β6 1:15 (10D5, MAB2077Z, Sigma-Aldrich) with same amount of normal rabbit IgG (2729, Cell Signaling Technology, Danvers, MA, USA) as control IgG, or the selective TGFβ type I receptor inhibitor GW788388, 10 µmol/l (SML0116, Sigma-Aldrich, solved in DMSO) with DMSO as vehicle control. After treatment for 24 hours, cardiac slices were washed in HBSS on inserts and processed further for immunostaining, RNA isolation and Western blot analysis as described.

Dissociation assay

Cells were treated as indicated and grown to confluency in 24-well plates. Cell monolayers were washed with HBSS and incubated with dissociation buffer (dispase II 2.5 U/mL, Sigma-Aldrich, D4693 in HBSS) at 37 °C till detachment of the cell monolayer from well bottom. After detachment, monolayers were mechanically stressed by defined pipetting using an electrical pipette (Eppendorf, Hamburg, Germany). The total number of resulting fragments per well was determined using a binocular stereo microscope (SZX2, Olympus). Fragments were counted if they were

clearly visible at 1.25-fold magnification. The number of fragments is an indirect measure for intercellular cohesion. Images were acquired with a SLR camera (EOS 800D, Canon).

Western blot analysis

Western blot analysis was performed using standard procedures. Tissue samples were homogenized in SDS-lysis buffer (12.5 mmol/l HEPES, 1 mmol/l EDTA, 12.5 mmol/l sodium fluoride, 0.5 % sodium dodecyl sulfate, pH 7.6) supplemented with protease inhibitor cocktail (cOmplete) and phosphatase inhibitor cocktail (PhosSTOP, both Roche, Basel, Switzerland) using FastPrep-24 5G bead beating grinder (MP Biomedicals, Santa Ana, CA, USA) and subsequently cleared by centrifugation. Confluent cell monolayers were washed with PBS and scraped in supplemented SDS lysis buffer. Lysates were sonicated and the total protein amount was determined with a BCA protein assay kit (Thermo Fisher Scientific) according to the manufacturer's instructions. After lysates were denaturized for 5 minutes at 95 °C in Lämmli buffer, gel electrophoresis and wet blotting on nitrocellulose membranes (Novex, Thermo Fisher Scientific) were performed according to standard procedures. After a drying step, membranes were blocked in Intercept blocking buffer (Li-Cor, Lincoln, NE, USA) diluted 1:1 in TBS for 1 hour at room temperature. The following primary antibodies were incubated in antibody buffer (Intercept blocking buffer diluted 1:1 in TBS containing 0.2 % tween 20) at 4 °C overnight: Mouse anti-DSG1/2 (61002, Progen, Heidelberg, Germany), mouse anti-DSP (61003, Progen), mouse anti-PG (61005, Progen), mouse anti-N-Cadherin (NCAD, 610921, BD Bioscience, Franklin Lakes, NJ, USA), mouse anti-PKP2 (651101, Progen), rabbit anti-ITGB6 (HPA023626, Sigma-Aldrich), mouse anti-β-catenin (BCAT, 610154, BD Bioscience), rabbit anti-GAPDH (10494-1-AP, Proteintech, Rosemont, IL, USA), mouse anti-α-tubulin (ab7291, Abcam, Cambridge, UK), rabbit anti-ITGAV (ab179475, Abcam), mouse anti-vinculin (VCL,

V9264, Sigma-Aldrich), rabbit anti-talin-2 (TLN2, MA5-42740, Thermo Fisher Scientific).

The secondary antibodies goat anti-mouse 800CW (925-32210) and goat anti-rabbit 680RD (925-68071, both Li-Cor) were incubated in TBS containing 0.1 % tween20 for 1 hour. Odyssey FC imaging system was used for fluorescence-based detection. Median band density was quantified applying ImageStudio (both Li-Cor) according to manufacturer's instructions and normalized to the respective loading control.

Immunostaining

For cryosections, tissue was embedded in 12 % mowiol 4-88, 5 % sorbitol, 0.5 % bovine serum albumin, 0.025 % sodium azide and frozen at -50 °C. 10 µm thick section were cut by a Cryo Star NX70 cryostat (Thermo Fisher Scientific), transferred to SuperFrost plus glass slides (Thermo Fisher Scientific), and air-dried. For immunostaining, sections were dried at 37 °C for 30 min, fixed in 2 % paraformaldehyde in PBS for 10 min, permeabilized with 0.2 % triton X-100 in PBS for 1 hour, and blocked with 3 % bovine serum albumin/0.12 % normal goat serum in PBS for 1 hour.

Fixed cardiac tissue embedded in paraffin was cut into 5 µm thick sections by an automated microtome (HM355S, Thermo Fisher Scientific). After deparaffinization, temperature-mediated antigen retrieval was performed in Tris/EGTA buffer (10 mmol/l Tris, 1 mmol/l EGTA, 0.05 % tween 20, pH 9) for 20 min at 95 °C. Tissue was permeabilized in 0.1 % triton X-100 in PBS for 5 min and blocked with 3 % bovine serum albumin/0.12 % normal goat serum in PBS for 1 hour.

The following primary antibodies were incubated in PBS at 4 °C overnight: Mouse anti-DSG1/2 (61002, Progen), mouse anti-DSP (61003, Progen), mouse anti-PG (61005, Progen), mouse anti-desocollin-2/3 (DSC2/3, 326200, Thermo Fisher Scientific), mouse anti-N-cadherin (NCAD, 610921, BD Bioscience), rabbit anti-ITGB6

(HPA023626, Sigma-Aldrich), rabbit anti-ITGB6/AV (BS-5791R, Bioss, Woburn, MA, USA), rabbit anti-ITGB1 (GTX112971, GeneTex, Irvine, CA, USA), rabbit anti-pSMAD2(S465/S467)/pSMAD3(S423/S425) (AP0548, Abclonal, Wuhan, China), rabbit anti-connexin-43 (CX43, SAB4501175, Sigma-Aldrich), rabbit anti-ITGAV (ab179475, Abcam), mouse anti-vinculin (VCL, V9264, Sigma-Aldrich), rabbit anti-talin-2 (TLN2, MA5-42740, Thermo Fisher Scientific). Respective secondary goat anti-rabbit or goat anti-mouse antibodies coupled to Alexa Fluor 488, Alexa Fluor 568 (both Thermo Fisher Scientific), or cy5 (Dianova, Hamburg, Germany) were incubated for 1 hour at room temperature and DAPI (Sigma-Aldrich) was added for 10 minutes to counterstain nuclei. To visualize F-actin, phalloidin coupled to Dylight 488 (21833, Thermo Fisher Scientific) or CruzFluor 647 (sc-363797, Santa Cruz Biotechnology, Dallas, TX, USA) was used. Tissue samples were mounted with Fluoromount Aqueous Mounting Medium (Sigma-Aldrich). For wide field image acquisition, a 40x objective mounted on a Nanozoomer S60 slide scanner (Hamamatsu Photonics K.K., Hamamatsu, Japan) and for confocal image acquisition a 63x PL APO NA = 1.4 objective mounted on a LSM710 confocal microscope (Carl Zeiss, Jena, Germany) or a 63x HCX Plan-Apo NA = 1.4 objective mounted on a Leica SP5 confocal microscope (Leica Biosystems) was used. Fluorescence image analysis was performed by Fiji/ImageJ (NIH) or QuPath (QuPath developers, The University of Edinburgh, UK, version 0.2.3). For analysis of staining intensity following masks and regions were defined: (i) “cardiomyocytes” – outline of the total cardiomyocyte area by thresholding of the f-acting signal, (ii) “nucleus” – detection of nuclei via DAPI staining applying the QuPath cell detection tool, (iii) “ICD” – outlines of ICD area were created with threshold of a respective counterstain or QuPath brush tool, (iv) “cytosol” - area of ICD was subtracted from cardiomyocyte area. Respective masks were applied to the channel

of interest to measure mean nuclear intensity in selected areas. The corresponding mean signal background was subtracted for ICD intensity.

Histological staining

Tissue was embedded and cut as described in the section *Immunostaining*. Haematoxylin/ Eosin (HE) staining was performed according to standard procedures. In brief, sections were stained with Mayer's haemalaun solution (Sigma-Aldrich) for 5 min, washed, dehydrated in an increasing ethanol series and stained with 0.5 % (w/v) eosin solution for 5 min. After washing steps in ethanol and methyl salicylate, sections were mounted with DPX Mountant (Sigma-Aldrich).

For Picro-Sirius red collagen stain, sections were fixed in 2 % paraformaldehyde in PBS for 10 min. After washing in PBS and distilled water, sections were re-fixed in Bouin's solution (71 % picric acid, 24 % formaldehyde 37-40 %, 5 % glacial acetic acid) for 1 hour at 56 °C. Sections were then washed in distilled water and stained in Picro-Sirius Red Solution (SRS250, PSR-1-IFU, ScyTek laboratories Inc., Logan, UT, USA) for 1 hour. Staining reaction was stopped with 0.5 % acetic acid. After washing in 100 % ethanol, sections were cleared in methyl salicylate and mounted in DPX Mountant (Sigma-Aldrich).

Images of histological sections were acquired with a 40x objective mounted on a Nanozoomer S60 slide scanner (Hamamatsu Photonics K.K.) and visualized with the NDP.view software (version 2.7.43, Hamamatsu Photonics K.K.). The area of collagen was analysed by a blinded experimenter using the red fluorescence signal of collagen staining in QuPath software (QuPath developers). Areas of interest (i.e. right and left ventricles) were annotated and the total and fibrotic tissue area was classified according to the intensity threshold, which was calculated as 40 intensity values above the median intensity (for collagen- positive areas) and 25% of median intensity (for

total tissue area). For analysis of hypertrophy, the area of 20 cross-sectioned cardiomyocytes with a central nucleus was measured for every heart.

Protein purification

For protein expression, the Fc control construct or the extracellular domain of murine Dsg2-WT or Dsg2-W2A were cloned into the Fc-His-pEGFP-N3 vector as described above. Wildtype Chinese ovarian hamster cells (CHO) were transfected with the respective plasmids using TurboFect (Thermo Fisher Scientific) according to manufacturer's instructions and selected with geneticin (VWR) for two weeks. After stable cells were grown to confluency, the cell culture supernatant was collected, the proteinase inhibitors leupeptin, aprotinin, pepstatin and phenylmethylsulfonylfluoride (all VWR) added and remaining cells removed by centrifugation and filtration. The cleared supernatant was added to a column containing HisLink Protein Purification Resin (V8823, Promega) and his-tag fusion proteins were purified according to manufacturer's instructions. After imidazole-mediated elution, proteins were concentrated using Amicon Ultra-4 30 kDa centrifugal filter tubes (#UFC803024, Merck) and resuspended in sterile HBSS containing 1.2 mmol/l Ca^{2+}. Protein concentration was determined by BCA protein assay kit (Thermo Fisher Scientific) and purity confirmed by Western blot analysis as described above.

Single molecule force spectroscopy

For force spectroscopy experiments, a Nanowizard IV Atomic Force Microscope (AFM, JPK Instruments, Berlin, Germany) mounted on an inverted fluorescence microscope (IX83, Olympus) was used. Recombinant proteins were generated as described in the section *Protein purification*. Flexible Si3N4 AFM probes (MLCT cantilever, Bruker, Billerica, MA, USA) and mica surfaces (Grade V-4, 01874-CA, Structure Probe, Inc., West Chester, PA, USA) were coated with aldehyde-PEG_{20}-NHS ester spacer (BP-24296, Broadpharm, San Diego, CA, USA) to link recombinant molecules at a

concentration of 0.15 mg/mL as described in [43]. Force spectroscopy measurements were performed with the pyramid-shaped D-tip (nominal spring constant: 0.03 N/m) on functionalized mica sheets in HBSS containing 1.2 mmol/l Ca^{2+} and 0.1 % BSA at 37 °C. Spring constant was calibrated for each cantilever at 37 °C applying the thermal noise method. [44] Force spectroscopy experiments were performed in force mapping mode using following settings: relative setpoint 0.4 nN, z-length 0.3 – 0.5 µm, extend delay 0.1 s, pulling speed as indicated ranging from 0.5 µm/s to 15 µm/s, scanning area: 10 µm x 10 µm, 25 px x 25 px and recorded with the SPM Control v.4 software (JPK Instruments). Force distance blots were analysed using JPKSPM Data Processing software (version 6, JPK Instruments). For calculation of force histogram, extreme peak curve fit and application of Bell's equation, Origin software (Originlab, Northampton, MA, USA) was used.

Echocardiography and Electrocardiogram (ECG)

Transthoracic echocardiography was performed using the Vevo 2100 ultrasound system (VisualSonics, Toronto, ON Canada) equipped with a MS-550 linear-array probe working at a central frequency of 40 MHz. After the animals were anesthetized with 3.0 % (v/v) isoflurane carried by pure oxygen, they were placed at supine position on a pre-warmed imaging platform. Anesthesia was maintained by 1.5 % (v/v) isoflurane through a nose cone and the body temperature was controlled at around 37 °C by a rectal thermocouple probe. Eye gel (Lacrinorm) was applied to prevent ocular dehydration. Needle probes attached to ECG leads embedded in the imaging platform were inserted subcutaneously to each limb for ECG recording. Hairs on the chest were removed by applying commercially available hair removal cream (Nair). ECG was monitored during the whole procedure. LV geometry and function were evaluated using 2D guided M-mode at the mid-papillary muscle level from parasternal short-axis. LV anterior (LVAW) and posterior (LVPW) wall thickness and internal dimensions (LVID)

were measured from the M-mode during systole (s) and diastole (d). Values were averages of three cardiac cycles. Left ventricular ejection fraction (EF) was calculated from derived volumes (Vol), which are computed based on the Teichholz formula (LV Vol;d = (7.0 / (2.4 + LVID;d)) × LVID;d^3, LV Vol;s = (7.0 / (2.4 + LVID;s)) × LVID;s^3, EF % = 100 × ((LV Vol;d – LV Vol;s) / LV Vol;d)). Left ventricular mass (LV Mass) was calculated based on a corrected cube model (LV Mass = 1.053 × ((LVAW;d + LVID;d + LVPW;d)^3 – LVID;d^3) × 0.8). Pulse-Wave (PW) Doppler imaging and tissue Doppler imaging in the apical four-chamber view were used to record mitral Doppler flow spectrum and mitral annulus velocity at the septal side, respectively. Peak blood velocity of mitral early filling (E), later atrial contraction (A), isovolumic contraction time (IVCT), isovolumic relaxation time (IVRT), aortic ejection time (AET), and peak annulus velocity at early diastole (E') were measured. Ratios of E/A, E/E' and Tei index (Tei index = (IVCT + IVRT) / AET) were calculated. Blood flow through the pulmonary artery was recorded with PW Doppler imaging in the parasternal long-axis view for the measurements of pulmonary acceleration time (PAT) and pulmonary ejection time (PET). The ratio of PAT/PET was calculated. To assess RV function, the dimensional changes of the RV outflow tract at aortic valve level were recorded by M-mode in the parasternal long axis view and calculated as RV FS. RV fractional area change (RV FAC) was measured with B-mode at the mid-papillary level in the parasternal short-axis view. Briefly, the RV areas at end-diastole (RV Area;d) and end-systole (RV Area;s) were measured and RV FAC was calculated as 100 × (RV Area;d – RV Area;s) / RV Area;d (%). Tricuspid annular plane systolic excursion (TAPSE) was measured in M-mode on the lateral tricuspid annulus near the RV free wall in the apical four-chamber view. Data was transferred to an offline computer and analyzed with Vevo 2100 software (version 1.6.0, VisualSonics) by an investigator blinded to the study groups.

For ECG recording, mice were attached to the PowerLab Data Acquisition System (ML870 Powerlab 8/30, ADInstruments, Sydney Australia). Needle probes were inserted subcutaneously in the right upper, and both lower limbs for acquisition of lead II. Baseline ECG was recorded for 30 min.

ECG data were recorded and analysed using the LabChart Pro 8 software (ADInstruments) equipped with the ECG Analysis Module. Peak amplitudes and intervals were determined from a curve averaged from 50 subsequent QRS complexes as mean of three time points. Definition of peaks is shown in Figure 2I.

After final measurements, mice were euthanized via cervical dislocation under anethesia and hearts were dissected. Organs were embedded and stained as described in the section *Histological staining* for further analysis to determine the amount of fibrosis. For wet/dry ratio, weight of the isolated lungs was determined before and after drying the tissue for 24 hours at 37 °C.

Transmission Electron Microscopy (TEM)

Ventricular cardiac tissue was dissected, cut and fixed in 2 % paraformaldehyde and 2.5 % glutaraldehyde (Electron Microscopy Sciences, Hatfield, PA, USA) in 0.1 mol/l Pipes, 2 mmol/l $CaCl_2$, pH 7.3. After 15 min incubation, fixative was renewed and incubated at 4 °C for 16 hours. Samples were washed three times with cold 0.1 mol/l Pipes, 2 mmol/l $CaCl_2$, pH 7.3 and rinsed with 0.1 mol/l cacodylate buffer, pH 7.3. Post-fixation was performed for 1 hour at 4 °C using 1 % osmium tetroxide and 0.8 % potassium ferracyanide (Electron Microscopy Sciences) in 0.1 mol/l cacodylate buffer, pH 7.3. After washing steps with cacodylate buffer, pH 7.3 and ultrapure distilled water, tissue samples were stained with 1 % aqueous uranyl acetate (Electron Microscopy Sciences) for 1 hour at 4 °C. Dehydration was performed by an ascending ethanol series at 4 °C. After three washes with 100 % ethanol, samples were rinsed in acetone and first embedded in a mixture of resin/acetone followed by pure Epon 812 resin

(Electron Microscopy Sciences) over night. Samples were mounted on BEEM capsules (Electron Microscopy Sciences) filled with EPON. After polymerization at 60 °C for 48 hours, samples were removed from the EPON block with the nitrogen hot water method. 70 nm thin serial sections, cut with a diamond knife, were mounted on formvar-carbon coated copper slot grids, stained with uranyl acetate and Reynolds's lead citrate. Samples were examined in a FEI Tecnai T12 spirit Transmission Electron Microscope (Thermo Fisher Scientific) operating at 80 kV equipped with a CCD Veleta digital camera.

RNA isolation

Cardiac tissue was washed in ice-cold HBSS and lysed in TRI reagent (Molecular Research Center, Inc., Cincinnati, OH, USA). Tissue homogenization was conducted via the FastPrep-24 5G bead beating grinder (MP Biomedicals, Santa Ana, CA, USA) using 2.8 mm stainless steel beads (Sigma-Aldrich) according to manufacturer's protocol with subsequent centrifugation to clear the lysate. RNA was isolated via the Direct-zol RNA MiniPrep kit including Zymo-Spin II and DNAse restriction step (R2050, Zymo research, Irvine, CA, USA).

Quantitative Real time PCR (qRT-PCR)

RNA was isolated as described above. Quantity and quality of RNA was determined by Nanodrop 1000 Spectrophotometer (Thermo Fisher Scientific). Up to 1 µg of isolated RNA was used for reverse transcription with SuperScript III (Thermo Fisher Scientific). Quantitative real time PCR was performed with StepOne Real time PCR Systems (Applied Biosystems) using Power SYBR Green PCR Master Mix (Thermo Fisher Scientific). Primers are listed in Table S3. As reference, the mean Ct value of *Gapdh* and *Tubg2* of the respective sample was used.

Mouse RNA-sequencing (RNA-Seq)

For transcriptomic analysis before and after onset of fibrosis, hearts of 5-days- and 9-weeks-old mice were dissected. Wt/wt and mut/mut mice were matched for age and sex. For 5-days-old mice, atria were removed and both ventricles lysed. For 9-weeks-old animals, similar sized tissue samples were taken from the right and left ventricle via a 3 mm diameter biopsy punch (Viollier, Allschwil, Switzerland). RNA was isolated as described in the section *RNA isolation*. RNA samples were quality-checked on the TapeStation instrument (Agilent Technologies, Santa Clara, CA, USA) using the RNA ScreenTape (Agilent, #5067-5576). RNA were quantified by Fluorometry using the QuantiFluor RNA System (#E3310, Promega). Library preparation was performed, starting from 200ng total RNA, using the TruSeq Stranded mRNA Library Kit (#20020595, Illumina, San Diego, CA, USA) and the TruSeq RNA UD Indexes (#20022371, Illumina). 15 cycles of PCR were performed. Libraries were quality-checked on the Fragment Analyzer (Advanced Analytical, Ames, IA, USA) using the Standard Sensitivity NGS Fragment Analysis Kit (#DNF-473, Advanced Analytical) revealing excellent quality of libraries (average concentration was 179±9 nmol/l and average library size was 329±3 base pairs). For Sequencing, samples were pooled to equal molarity. The pool was quantified by Fluorometry using the QuantiFluor ONE dsDNA System (#E4871, Promega). Libraries were sequenced Paired-End 38 bases (in addition: 8 bases for index 1 and 8 bases for index 2) using the NextSeq 500 High Output Kit 75-cycles (#FC-404-1005, Illumina) loaded at 1.8pM and including 1 % PhiX. Primary data analysis was performed with the Illumina RTA version 2.11.3. On average per sample: 38.7±4.6 million pass-filter reads were collected on that NextSeq 500 Flow-Cell.

Mouse RNA-Seq data analysis

Reads were aligned to the mouse mm10 genome using the aligner STAR (version 2.7.3a) [45] with extra options "--outFilterMultimapNmax 10 --outSAMmultNmax 1" for

handling multimapping reads. Aligned reads were assigned to ensembl genes (version 101) using the tool featureCounts from the subread package (version 2.0.1) [46] with extra options "-O -M --read2pos5 –primary-s 2". All further analysis steps were performed with R/Bioconductor (R version 4.0.3, Bioconductor version 2.50.0). Gene counts were loaded into R and differential gene expression analysis followed the edgeR workflow [47]. Specifically, genes were filtered for expression using the function filterByExpr which retained 19504 genes. Samples were classified into 4 groups according to genotype (wt/wt and mut/mut) and time point (5 days and 9 weeks) and differential gene expression was performed between all 4 groups using the functions glmQLFit and glmQLFTest. Benjamini-Hochberg procedure was performed to correct for multiple testing. Principal component analysis (PCA) was performed using log-CPM values and applying the R prcomp function on the row- and column-centered expression matrix. Sample clustering within heatmaps uses a correlation-based distance and employs the complete linkage algorithm of the R function hclust. Gene set enrichment analysis (GSEA) was performed for all differentially expressed genes of the indicated condition and compared to the collections of gene sets form MSigDb (https://www.gseamsigdb.org/gsea/msigdb/collections.jsp) or selected gene sets as indicated and relied on the edgeR function camera. External gene lists were tested on different contrasts using the cameraPR function of edgeR. Indicated p-values were calculated by function cameraPR of the R package limma (R version 4.0.3, Bioconductor version 2.50.0) using the log-fold-change as the input statistics and employing a rank based test. P-values are adjusted for multiple testing using the Benjamini-Hochberg procedure.

Re-analysis of available ACM data sets and comparison with murine data sets

Raw data of GEO data sets GSE107157 and GSE107480 were downloaded from the European Nucleotide Archive and mapped to the human hg38AnalysisSet genome

using STAR. Gene expression was quantified with featureCounts and relied on the human ensembl gene annotation (version 96). Gene counts of both data sets were imported into R and analysed using the edgeR workflow. Specifically, genes were filtered by expression which retained 25293 genes, followed by a differential expression analysis contrasting the 4 sample groups which arise from tissue source (left and right ventricle) and disease (ARVC and healthy). In order to compare mouse specific gene lists to the human data, mouse genes were mapped to human orthologs using BioMart. The gene set of human orthologs was tested for differential enrichment in the mouse gene expression contrasts defined above using the function cameraPR. For analysis of the GEO data set GSE29819, the GEO2R tool was used (ACM samples versus normal). Results with adjusted p-values<0.05 were considered as significantly changed. The Venn diagram for overlap between two groups was determined with the Venny 2.1.0 online tool. Courtesy: Oliveros, J.C. (2007–2015) Venny. An interactive tool for comparing lists with Venn's diagrams. Publicly available at http://bioinfogp.cnb.csic.es/tools/venny/index.html.

Fluorescence recovery after photobleaching (FRAP)

For FRAP measurements, neonatal cardiomyocytes cells were isolated from mice according to the protocol of Ehler et al. [48] and seeded in eight-well imaging chambers (Ibidi, Martinsried, Germany). 24 hours after seeding, the cells were transduced with DSG2-WT-mGFP or DSG2-W2A-mGFP lentivirus as described above. After the formation of visible junctions, FRAP measurements were performed on a Stellaris 8 Falcon confocal microscope (Leica, Wetzlar, Germany) with a HC PL APO CS2 63x/1.40 Oil objective, at 37 °C with 5 % CO_2 and constant humidity. The measurements were carried out and analysed with the fluorescence recovery after photobleaching wizard software (Leica). In order to inhibit contractions of

cardiomyocytes, 40 mmol/l BDM was added shortly before FRAP experiment. Regions of interests were defined along cell-cell junctions containing a desmosome between two neighbouring mGFP positive cells. After five frames of recording the prebleach intensity, mGFP signal was bleached shortly for 3 frames, using the 488 nm laser line at 50 % transmission on FRAP booster mode and the fluorescence recovery was recorded over 239 s with 100 frames for the initial 26 s and 45 frames for the remaining time. The fraction of mobile molecules was determined by the formula: Mobile fraction = *Ie – Io/ Ii – Io*, where, *Ie* is the intensity reached after recovery time, *Io* is the minimal intensity that was achieved right after bleaching, and *Ii* is the average prebleach intensity value.

Triton X-100 protein separation

Tissue samples were lysed in Triton extraction buffer (0.5 % triton X-100, 50 mmol/l MES, 25 mmol/l EGTA, 2 mmol/l $MgCl_2$, pH 7.4) complemented with aprotinin, leupeptin, pepstatin, and phenylmethylsulfonyl fluoride by homogenization via the FastPrep-24 5G bead beating grinder (MP Biomedicals) using 2.8 mm stainless steel beads (Sigma-Aldrich). Samples were centrifuged at 12000 G, 5 min at 4 °C and cleared supernatant used as triton X-100 soluble fraction. The remaining pellet was lysed in SDS-lysis buffer and taken as triton X-100 non-soluble fraction. Protein concentration was determined using the BCA method (Thermo Fisher Scientific). The samples were loaded on SDS polyacrylamide gels and Western blot analysis was performed as described above. Total protein stain was performed with Ponceau stain (5 % glacial acetic acid, 0.1 % Ponceau S red dye) for 5 min. After de-stain with water, bands were acquired in the 700 nm channel. For normalization, intensity of the entire lane was taken.

Structured illumination microscopy (SIM)

For structured illumination microscopy (SIM) of cryopreserved mouse heart tissue, sectioning and immunostaining were performed as described. Image stacks spanning full ICDs were acquired using a DeltaVision OMX-Blaze (Version 4; Applied Precision) equipped with a 60x PL APO NA = 1.42 objective (Olympus) yielding a voxel size of 0.04 x 0.04 x 0.125 µm.

For analysis, the observer was generally blinded for the genotype. For evaluation of signal size and frequency within ICDs, the image stacks (typically containing one clearly defined ICD) were loaded into Imaris 9.6 (Bitplane AG, Schlieren, Switzerland). The ICD was annotated by manually outlining the DSP signal in every third image plane. The outlines were converted into a volume using the Manual Surface module. The Euclidian distance between two extreme ends of the surface was denoted as ICD length. To visualize the distribution of DSG2 and ITGB6 within the ICD, the signals were automatically detected and annotated as individual objects using similar thresholding values. The Statistics module was applied to calculate amounts and spatial relationships of signals.

2. Supplemental Tables

	wt/wt	mut/mut	*mut/mut vs. wt/wt*	wt/wt	mut/wt	mut/mut	*mut/mut vs. wt/wt*	wt/wt	mut/wt no fibrosis	mut/wt with fibrosis	*mut/wt with fibrosis vs. wt/wt*
Mean age:	4 months			6 months				12 months			
**General**											
wet/dry lung weight				4.38 ±0.33		4.36 ±0.16	*ns*	4.59 ±0.10	4.53 ±0.10	4.55 ±0.10	*ns*
Body weight (g)	25.25 ±4.87	22.28 ±2.88	*ns*	29.22 ±6.41	28.90 ±3.78	26.68 ±3.81	*ns*	34.43 ±4.09	35.49 ±6.16	34.53 ±6.62	*ns*
**Echocardiography**											
Heart rate (bpm)	432.50 ±88.13	488.00 ±27.20	*ns*	420.42 ±49.04	385.83 ±36.43	455.00 ±70.44	*ns*	418.07 ±46.23	428.77 ±55.97	448.50 ±63.77	*ns*
Right ventricle											
PAT/PET	0.30 ±0.03	0.23 ±0.05	*0.15*	0.33 ±0.04	0.29 ±0.04	0.23 ±0.06	*	0.34 ±0.07	0.32 ±0.05	0.31 ±0.04	*ns*
TV S'-Vel (mm/s)	39.29 ±7.86	19.50 ±1.01	*	25.14 ±3.42	22.72 ±5.46	21.96 ±4.87	*ns*	0.92 ±0.15	23.82 ±3.96	19.76 ±6.65	*0.11*
wallthickness (mm)	0.27±0.09	0.27 ±0.05	*ns*	0.28 ±0.04	0.26 ±0.05	0.29 ±0.08	*ns*	0.28 ±0.06	0.29 ±0.06	0.31 ±0.10	*ns*
FAC (%)	37.43 ±9.44	17.48 ±6.24	*	36.64 ±11.41	31.89 ±7.92	20.64 ±7.26	*0.15*	37.49 ±7.95	38.58 ±7.81	28.02 ±9.42	*0.10*
Left ventricle											
LVAW;d (mm)	0.74 ±0.10	0.72 ±0.09	*ns*	0.72 ±0.11	0.77 ±0.10	0.65 ±0.19	*ns*	0.82 ±0.13	0.86 ±0.13	0.81 ±0.13	*ns*
LVID;d (mm)	4.36 ±0.30	4.32 ±0.26	*ns*	4.19 ±0.38	4.08 ±0.11	4.67 ±0.52	*	4.27 ±0.33	4.10 ±0.30	4.04 ±0.42	*ns*
LVPW;d (mm)	0.75 ±0.12	0.71 ±0.12	*ns*	0.73 ±0.11	0.69 ±0.07	0.70 ±0.13	*ns*	0.78 ±0.09	0.82 ±0.15	0.80 ±0.12	*ns*
LVAW;s (mm)	1.09 ±0.08	0.97 ±0.16	*ns*	1.05 ±0.18	1.15 ±0.19	0.74 ±0.25	*	1.16 ±0.17	1.20 ±0.19	1.18 ±0.21	*ns*
LVID;s (mm)	3.10 ±0.25	3.26 ±0.44	*ns*	3.04 ±0.51	2.89 ±0.28	4.05 ±0.56	*	3.10 ±0.40	2.93 ±0.31	2.79 ±0.28	*ns*
LVPW;s (mm)	1.11 ±0.18	1.05 ±0.17	*ns*	1.07 ±0.17	1.04 ±0.12	0.92 ±0.12	*0.08*	1.11 ±0.14	1.17 ±0.20	1.20 ±0.20	*ns*
FS (%)	28.94 ±3.49	24.83 ±6.34	*ns*	27.98 ±6.15	29.19 ±6.19	13.48 ±5.11	*	27.52 ±5.48	28.58 ±4.15	30.16 ±4.22	*ns*
SV (µl)	48.35 ±7.37	38.74 ±3.85	*ns*	40.60 ±5.73	40.63 ±4.81	28.43 ±11.05	*	44.25 ±9.08	41.51 ±7.49	41.36 ±9.91	*ns*
Output (ml/min)	21.00 ±5.43	18.92 ±2.32	*ns*	16.99 ±2.53	15.75 ±2.98	12.61 ±3.99	*	18.48 ±4.37	17.74 ±3.82	18.53 ±4.82	*ns*
MV A-Vel (mm/s)	469.34 ±152.33	268.79 ±101.26	*	408.56 ±105.75	336.92 ±85.23	165.00 ±90.18	*	423.37 ±128.18	367.8 ±91.59	418.3 ±90.78	*ns*
MV E-Vel (mm/s)	760.10 ±136.72	628.19 ±222.32	*ns*	600.81 ±145.35	570.44 ±86.71	532.94 ±72.21	*ns*	608.62 ±111.32	576.02 ±127.21	573.78 ±63.13	*ns*
Tei-index	0.41 ±0.08	0.43 ±0.09	*ns*	0.65 ±0.12	0.47 ±0.07	0.60 ±0.28	*ns*	0.49 ±0.16	0.53 ±0.14	0.44 ±0.07	*ns*
MV E/A	1.68 ±0.31	2.48 ±1.13	*ns*	1.50 ±0.31	1.74 ±0.25	4.14 ±2.25	*0.15*	1.54 ±0.46	1.69 ±0.67	1.40 ±0.13	*ns*
MV E/E'	-40.68 ±14.69	-38.37 ±5.12	*ns*	-36.74 ±4.66	-32.92 ±3.80	-39.33 ±15.56	*ns*	-38.99 ±7.97	-35.93 ±9.84	-45.30 ±10.46	*ns*
LV mass/ BW (mg/g)	3.96 ±0.37	4.19 ±0.68	*ns*	3.12 ±0.64	2.98 ±0.24	3.78 ±1.18	*ns*	3.10 ±0.67	3.02 ±0.72	2.84 ±0.54	*ns*
**ECG**											
Heart rate (bpm)	471.5 ±46.89	514.1 ±27.97	*ns*	420.8 ±38.55	413 ±30.21	470.5 ±75.79	*0.09*	433.5 ±48.15	454.4 ±65.94	458.7 ±31.21	*ns*
PR interval (ms)	39.47 ±0.62	42.69 ±5.63	*ns*	43.64 ±3.20	41.94 ±1.55	43.3 ±6.62	*ns*	42.55 ±4.55	42.75 ±3.48	41.24 ±1.07	*ns*
P amplitude (mV)	0.112 ±0.029	0.058 ±0.092	*	0.089 ±0.023	0.109 ±0.031	0.068 ±0.017	*ns*	0.102 ±0.017	0.091 ±0.020	0.095 ±0.023	*ns*
Q amplitude (mV)	0.008 ±0.005	-0.018 ±0.026	*	0.010 ±0.010	0.004 ±0.015	-0.012 ±0.025	*	0.012 ±0.005	0.013 ±0.014	0.012 ±0.011	*ns*
R amplitude (mV)	1.18 ±0.03	0.83 ±0.29	*0.06*	0.81 ±0.22	0.78 ±0.10	0.65 ±0.26	*0.2*	0.73 ±0.18	0.69 ±0.23	0.58 ±0.07	*ns*

Table S1. Detailed analysis of the cardiac phenotype in DSG2-W2A mice. Presented data correspond to echocardiography and ECG measurements in Figure 2 and include the same animals. Values indicate the mean ± standard deviation. For statistical comparison of all groups, distribution of data was analysed by Shapiro-Wilk normality test and group variance was analysed by Brown-Forsythe test. According to the results the respective test was applied: one-way ANOVA with Sidak's post hoc test for normal distributed data with similar variance, Welch's ANOVA for normal distributed data with different variance and Kruskal-Wallis test with Dunn's post hoc test for non-normal distributed data. Grey rows show the respective P values for the indicated comparisons with *P< 0.05 and *ns*P> 0.2. See detailed Methods section for abbreviations and calculation of parameters.

Category	mut/wt	mut/mut
**I. Morpho-functional ventricular abnormalities**	*Echocardiography:*<br>**RV systolic dysfunction** (RV FS) in hearts with RV fibrosis	*Echocardiography:*<br>**RV** and **LV systolic dysfunction** (TAPSE, RV FS, LV EF)
**II. Structural myocardial abnormalities**	*Histology:*<br>**RV fibrosis** occurring in 36% of mice age > 40 weeks	*Histology:*<br>**RV** and **LV fibrosis**
**III. Repolarization abnormalities**	*ECG:*<br>Tendency to reduced J amplitude	*ECG:*<br>Altered early repolarization with reduced **J amplitude**
**IV. Depolarization abnormalities**	*ECG:*<br>Reduction of **S amplitude**	*ECG:*<br>Elongation of **QRS interval**, reduction of **S amplitude**
**V. Ventricular arrythmias**	*ECG:*<br>Frequent **PVCs** in 78% of male mice with RV fibrosis	*ECG:*<br>Frequent **PVCs** in 56% of mice, one mouse with **non-sustained ventricular tachycardia**
**VI. Genetics**	**Likely pathogenic mutation** in desmosomal molecule	**Likely pathogenic mutation** in desmosomal molecule<br><br>Premature **sudden death**

Table S2. Comparison of the cardiac alterations in the DSG2-W2A mouse model to ACM diagnosis criteria applied in patients. Summary of data presented in Figure 2 and Table S1. Categorization according to the Padua ACM diagnosis criteria.[13]

Gene name	*Primer name*	*Sequence (5'→3')*	*Product size*
Col1a1	qPCR_mCol1a1_for	CCCAGCCGCAAAGAGTCTAC	152
Col1a1	qPCR_mCol1a1_rev	GGACCCTTAGGCCATTGTGT	
Lamc2	qPCR_mLamc2_for	GTGCCGGAGTTACCATCCAA	162
Lamc2	qPCR_mLamc2_rev	CAGACATCAAGGGCCGAAGT	
Fn1	qPCR_mFn1_for	CTGGATCCCCTCCCAGAGAA	193
Fn1	qPCR_mFn1_rev	TTGGGGTGTGGAAGGGTAAC	
Timp1	qPCR_mTimp1_for	AGATACCATGATGGCCCCCT	176
Timp1	qPCR_mTimp1_rev	TGGTCTCGTTGATTTCTGGGG	
Id2	qPCR_mId2_for	ACATCAGCATCCTGTCCTTGC	200
Id2	qPCR_mId2_rev	ACGTGTTCTCCTGGTGAAATGG	
Col3a1	qPCR_mCol3a1_for	CCAGTGGCCATAATGGGGAA	122
Col3a1	qPCR_mCol3a1_rev	ATCTCGACCTGGCTGACCAT	
Col1a2	qPCR_mCol1a2_for	TGGATACGCGGACTCTGTTG	87
Col1a2	qPCR_mCol1a2_rev	GGCCCTTTCGTACTGATCCC	
Itgb6	qPCR_mItgb6_for	TGGCACTTCTGCCAAAGACT	150
Itgb6	qPCR_mItgb6_rev	TTTCTGTCTGGGCTCACGTC	
Tubg2	qPCR_mTubg2_for	GGTCTGGGCTCCTACCTCTTA	96
Tubg2	qPCR_mTubg2_rev	ACTCATCTCGTCCTGGTTGG	
Gapdh	qPCR_mGapdh_for	CCCACTCTTCCACCTTCGAT	199
Gapdh	qPCR_mGapdh_rev	AGTTGGGATAGGGCCTCTCTT	

Table S3. Primers used in qRT-PCR experiments.

3. Supplemental Figures and Figure Legends

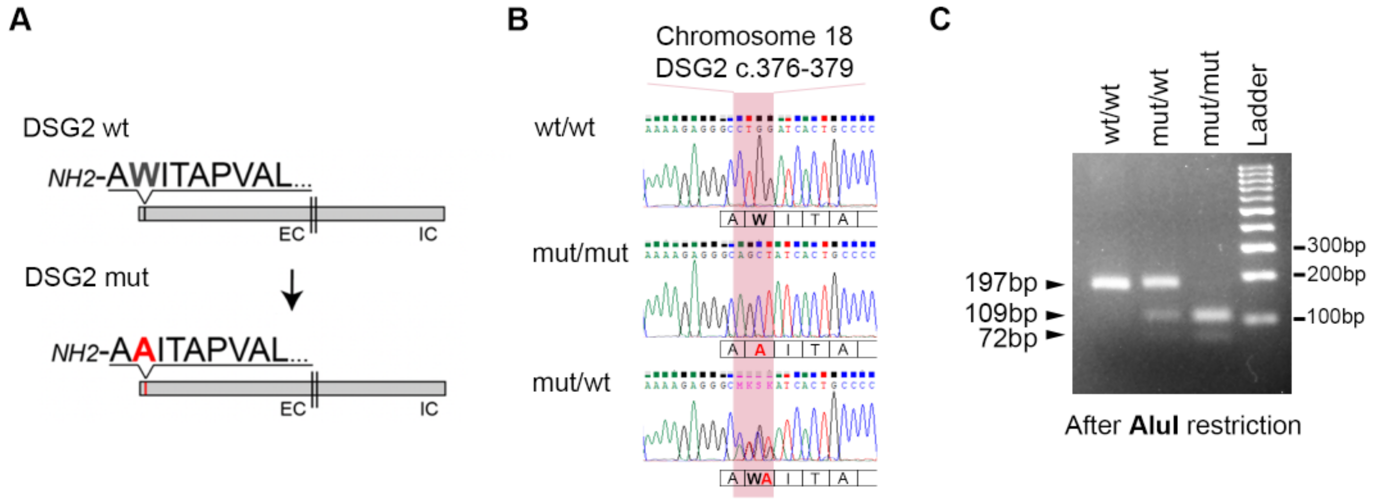

Figure S1. Sequencing and genotyping of the DSG2-W2A mouse model. (**A**) Schematic of the DSG2-W2A mutation within the mature DSG2 protein. The extracellular (EC) and intracellular (IC) part of the molecule is indicated. (**B**) Representative sequencing results of the DSG2-W2A mouse model for the indicated genotypes. The bars below show the related translation in amino acids. (**C**) Representative results of DSG2-W2A genotyping showing electrophoresis after restriction of the PCR product with AluI.

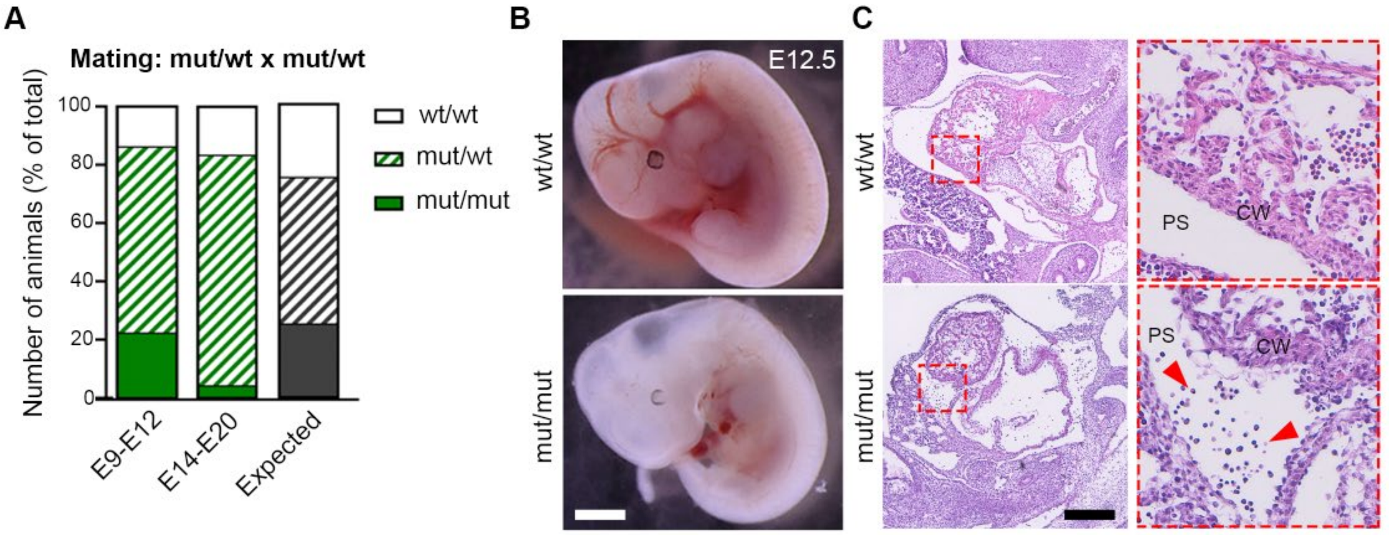

Figure S2. Loss of mut/mut animals during development due to pericardial bleeding. (**A**) Genotype analysis of embryos derived from matings of mut/wt animals between developmental day E9 to E20 compared to the expected genotype distribution according to Mendel in grey. (**B**) Macroscopic appearance of viable embryos at day E12.5. Scale bar: 2 mm. (**C**) Haematoxylin/eosin staining of sagittal sections of the cardiac area derived from embryos in **B**. Red rectangle depicts area of zoomed insert. Scale bar overview: 1 mm. Red arrowheads point to blood precursor cells in the pericardial space (PS) adjacent to the cardiac wall (CW). Images are representative for 4 embryos per genotype from 4 litters.

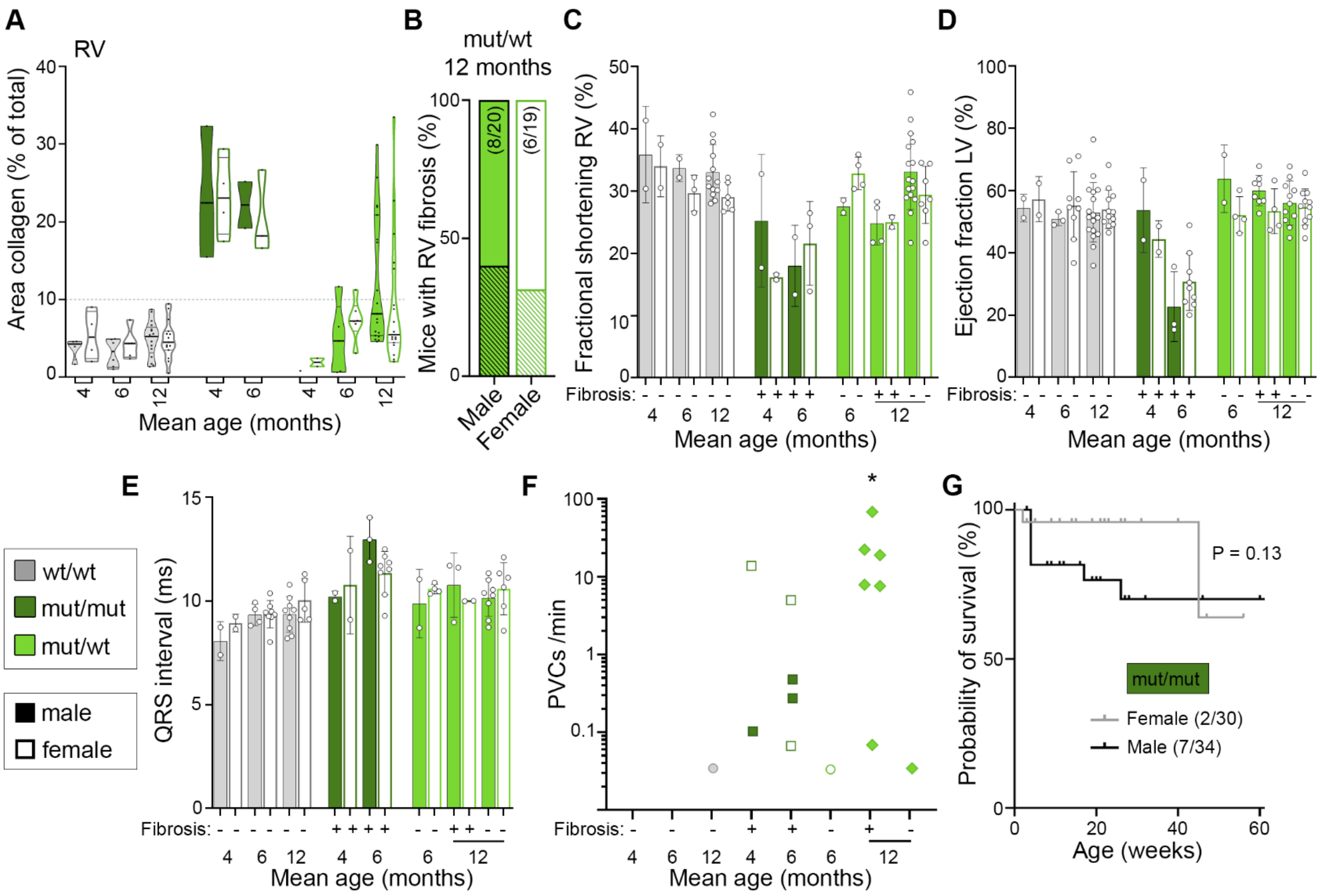

Figure S3. Sex-related effects in DSG2-W2A mice. Data presented in Figure 2 were re-analysed with respect to sex. (**A**) Cardiac fibrosis detected by picrosirius red collagen staining with analysis of the area of collagen in the right ventricle (RV). Hearts with more than 10% of collagen in the RV (grey dotted line) were defined as "with fibrosis". Each dot represents one animal. No significant changes comparing male vs. female, two-way ANOVA, Sidak's post hoc test. Lines indicate median and quartile values. (**B**) Analysis of the fraction of mut/wt mice presenting with RV fibrosis at 12 months of age. Values in bars indicate corresponding absolute number of mice with fibrosis (hatched bar). Ventricular function detected by echocardiography with analysis of (**C**) RV fractional shortening and (**D**) LV ejection fraction. No significant changes comparing male vs. female, two-way ANOVA, Sidak's post hoc test. (**E**) ECG recorded in lead II with analysis of QRS interval. No significant changes comparing male vs. female, two-way ANOVA, Sidak's post hoc test. (**F**) PVC burden detected by ECG and

depicted as number of PVCs per minute, *P< 0.05 male vs. female, two-way ANOVA, Sidak's post hoc test. (**G**) Kaplan-Meier survival diagram of DSG2-W2A mut/mut mice separated by sex. Vertical lines indicate drop-outs due to unrelated elimination (end of experiment, breeding, injuries). Values indicate corresponding absolute number of mice with sudden death compared to total number of mice evaluated. P as indicate, male vs. female. Gehan-Breslow-Wilcoxon test. Box with color indications of respective groups on the left apply to the entire figure.

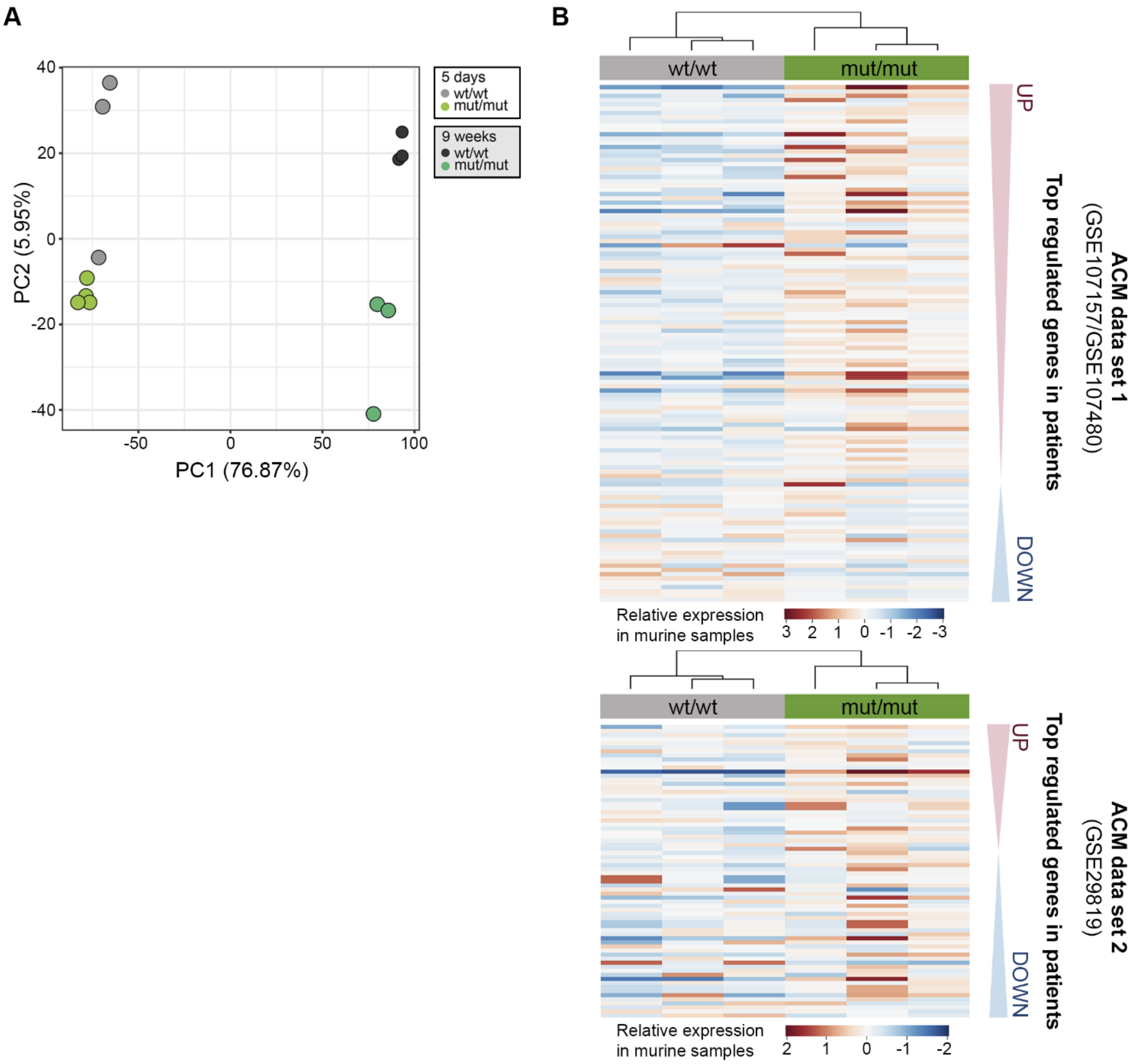

Figure S4. Validation of DGS2-W2A RNA-sequencing data. (**A**) Principal component analysis (PCA) of wt/wt and mut/mut hearts analysed by RNA sequencing at the age of 5 days and 9 weeks showing the first two principal components. (**B**) Heat maps of relative expression of the top differentially expressed genes derived from two ACM patient data sets (GEO: GSE107157/GSE107480[14] and GSE29819[15]) in 9-weeks-old mut/mut and wt/wt mouse hearts. Threshold of |Log fold change (FC)| > 2 and false discovery rate (FDR) < 0.05 was applied for gene selection. Samples are clustered based on their expression pattern. As depicted on the right, genes are

arranged according to their expression in patient data sets with most upregulated genes on top to most down regulated on the bottom.

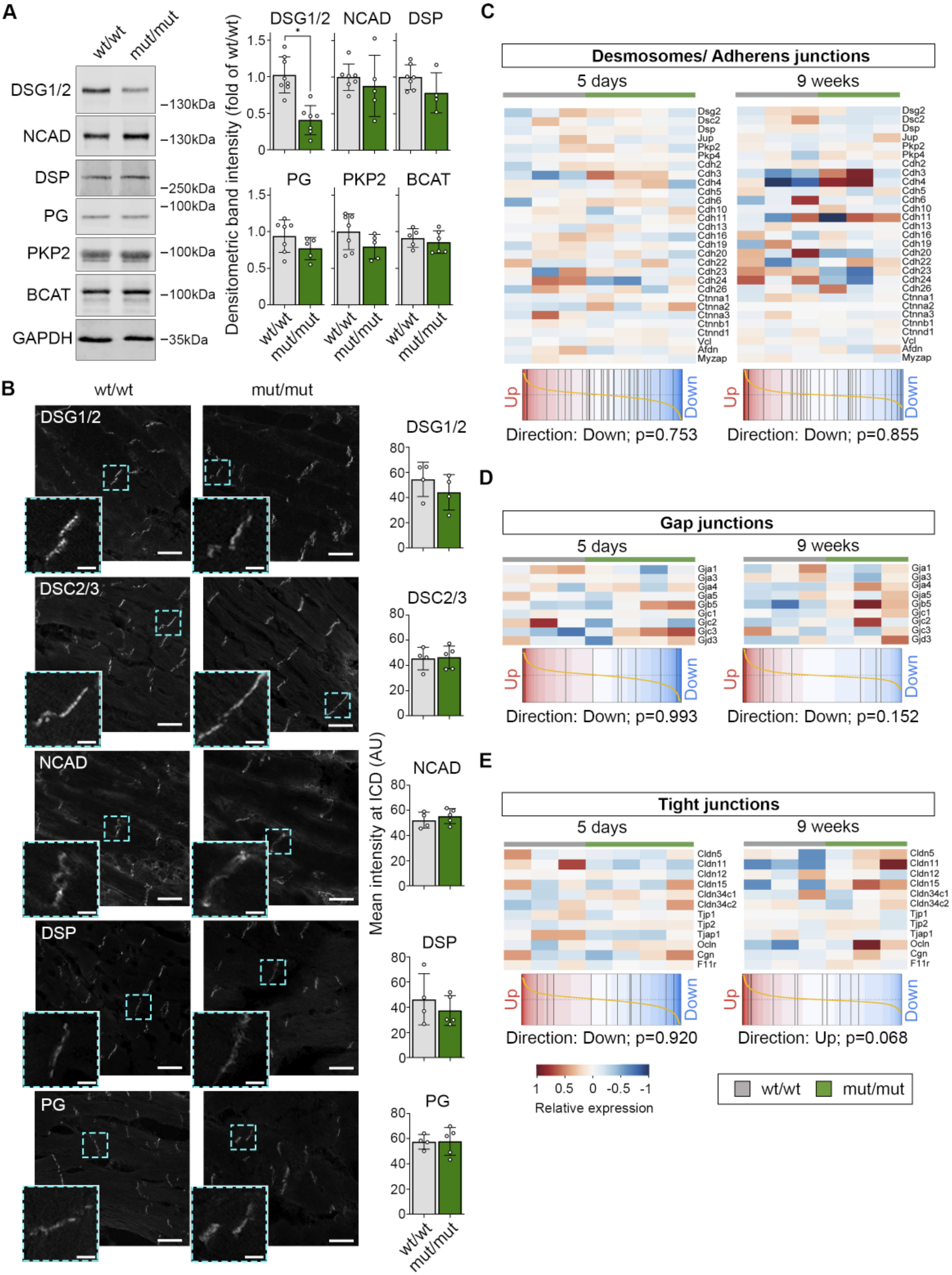
A
wt/wt
mut/mut
DSG1/2
NCAD
DSP
PG
PKP2
BCAT
GAPDH
−130kDa
−130kDa
−250kDa
−100kDa
−100kDa
−100kDa
−35kDa
Densitometric band intensity (fold of wt/wt)
B
DSC2/3
Mean intensity at ICD (AU)
C
Desmosomes/ Adherens junctions
5 days
9 weeks
Dsg2
Dsc2
Dsp
Jup
Pkp2
Pkp4
Cdh2
Cdh3
Cdh4
Cdh5
Cdh6
Cdh10
Cdh11
Cdh13
Cdh16
Cdh19
Cdh20
Cdh22
Cdh23
Cdh24
Cdh26
Ctnna1
Ctnna2
Ctnna3
Ctnnb1
Ctnnd1
Vcl
Afdn
Myzap
Up
Down
Direction: Down; p=0.753
Direction: Down; p=0.855
D
Gap junctions
Gja1
Gja3
Gja4
Gja5
Gjb5
Gjc1
Gjc2
Gjc3
Gjd3
Direction: Down; p=0.993
Direction: Down; p=0.152
E
Tight junctions
Cldn5
Cldn11
Cldn12
Cldn15
Cldn34c1
Cldn34c2
Tjp1
Tjp2
Tjap1
Ocln
Cgn
F11r
Direction: Down; p=0.920
Direction: Up; p=0.068
Relative expression
wt/wt
mut/mut

Figure S5. Junctional components are preserved in DSG2-W2A mutant hearts. (**A**) Western blot analysis and (**B**) immunostainings of adult DSG2-W2A wildtype and mutant hearts with representative blots or images, respectively, on the left and related analysis on the right. Desmoglein-1/2 (DSG1/2), Desmocollin-2/3 (DSC2/3), N-cadherin (NCAD), desmoplakin (DSP), plakoglobin (PG), plakophilin-2 (PKP2) β-catenin (BCAT) were analysed as components of the mechanical junctions at the ICDs. GAPDH served as loading control in **B**. *P< 0.05, unpaired Student's t-test. Scale bars in **C**: overview 20 µm; insert 5 µm. Cyan rectangle depicts area of zoomed insert. (**C** - **E**) Heat maps of relative expression of genes belonging to the indicated junction type (genes indicated on the right) in 5-days- and 9-weeks-old mouse hearts. Gene set enrichment analyses of the included genes is depicted as barcode plot below. Samples are arranged according to their genotype (wt/wt: grey; mut/mut: green).

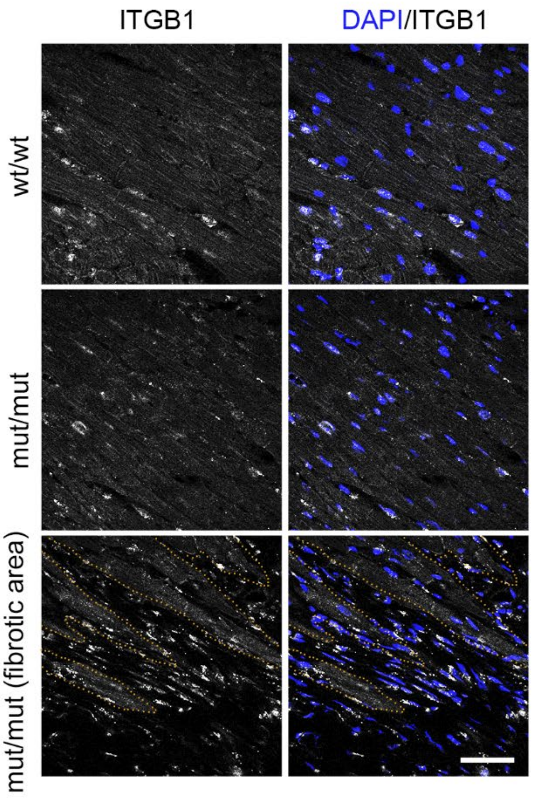

Figure S6. Integrin-β1 is not altered in DSG2-W2A mutant hearts. Integrin-β1 (ITGB1) immunostaining and nuclei counter stain (DAPI, blue) in DSG2-W2A mice. Lower row shows an overview image of a fibrotic area in mut/mut hearts. Dotted orange line highlights border of fibrotic tissue. Scale bar: 50 µm. Images representative for 5 mice per genotype.
